# Strong variation of spin-orbit torques with relative spin relaxation rates in ferrimagnets

**DOI:** 10.1038/s41467-023-37506-9

**Published:** 2023-03-30

**Authors:** Lijun Zhu, Daniel C. Ralph

**Affiliations:** 1 State Key Laboratory of Superlattices and Microstructures, Institute of Semiconductors, Chinese Academy of Sciences, Beijing, 100083 China; 2 College of Materials Science and Opto-Electronic Technology, University of Chinese Academy of Sciences, Beijing, 100049 China; 3 Cornell University, Ithaca, NY 14850 USA; 4 Kavli Institute at Cornell, Ithaca, NY 14850 USA

**Keywords:** Spintronics, Spintronics

## Abstract

Spin-orbit torques (SOTs) have been widely understood as an *interfacial* transfer of spin that is independent of the bulk properties of the magnetic layer. Here, we report that SOTs acting on ferrimagnetic Fe_*x*_Tb_1-*x*_ layers decrease and vanish upon approaching the magnetic compensation point because the rate of spin transfer to the magnetization becomes much slower than the rate of spin relaxation into the crystal lattice due to spin-orbit scattering. These results indicate that the relative rates of competing spin relaxation processes within magnetic layers play a critical role in determining the strength of SOTs, which provides a unified understanding for the diverse and even seemingly puzzling SOT phenomena in ferromagnetic and compensated systems. Our work indicates that spin-orbit scattering within the magnet should be minimized for efficient SOT devices. We also find that the interfacial spin-mixing conductance of interfaces of ferrimagnetic alloys (such as Fe_*x*_Tb_1-*x*_) is as large as that of 3*d* ferromagnets and insensitive to the degree of magnetic compensation.

## Abstract (web summary)

There has been a lot of interest in using antiferromagnets for magnetic memories, due to their fast dynamics, and resilience to stray fields. Such a memory was supposed to be switched by a spin-orbit torque. Here, Zhu and Ralph find that as a ferrimagnet approaches the magnetic compensation point, the spin-orbit torque acting on the ferrimagnet vanishes due to competing spin relaxation processes.

## Introduction

Efficient manipulation of magnetic materials is essential for spintronic devices. While spin-orbit torques (SOTs)^1,2^ are well established to be an effective tool to manipulate metallic 3*d* ferromagnets (FMs), whether they can effectively control antiferromagnetically-ordered systems has remained elusive despite the recent blooming of interest in ferrimagnets (FIMs) and antiferromagnets (AFs)^3–9^. Experimentally, for reasons unclear, the SOTs exerted on nearly compensated FIMs^5–7,10^ are often measured to be considerably weaker than those on 3*d* FMs for a given spin-current generator (by up to >20 times, see below). More strikingly, it remains under debate whether perfectly compensated FIMs and collinear AFs (*M*_s_ = 0 emu/cm^3^) can be switched at all by SOTs^11–13^.

Microscopically, SOTs have been widely assumed as an interfacial transfer of spin (i.e., spin dephasing length *λ*_dp_ ≈ 0 nm for transverse spin current) that is independent of the bulk properties of the magnetic layer, such as in drift-diffusion analyses^14–16^. Under this assumption, spin current entering the magnet from an adjacent spin-generating layer is absorbed fully by the magnetization via dephasing to generate SOTs, and the damping-like SOT efficiency per current density (${\xi }_{{DL}}^{j}$) will depend only on the spin Hall ratio (*θ*_SH_) of the spin-generating layer and the spin transparency (*T*_int_) of the interface which determines what fraction of the spin current enters the magnet^17,18^, i.e.,

1 $${\xi }_{{DL}}^{j}={T}_{{{{{{\rm{int}}}}}}}{\theta }_{{{{{{\rm{SH}}}}}}}.$$

This picture is a reasonable approximation for sufficiently thick metallic FMs that have a short *λ*_dp_ (≤1 nm) due to strong exchange coupling^19–21^ and a long spin diffusion length (*λ*_s_) associated with spin relaxation due to spin-orbit scattering^22,23^. However, in antiferromagnetically-ordered systems *λ*_dp_ can be quite long, as predicted more than a decade ago^24–27^, which, as discussed below, questions the widely accepted models of “interfacial torques”, particularly, in FIMs with strong spin-orbit scattering. So far, any roles of the bulk properties of the magnetic layer, e.g., the competing spin relaxation rates, in the determination of ${\xi }_{{DL}}^{j}$ have been overlooked in SOT analyses.

Here, we report measurements of SOTs acting on ferrimagnetic Fe_*x*_Tb_1-*x*_ layers with strong spin-orbit coupling (SOC)^8^ by tuning the Fe_*x*_Tb_1-*x*_ composition and temperature (*T*). We find that, in contrast to the prediction of Eq. (1), ${\xi }_{{DL}}^{j}$varies strongly with the degree of magnetic compensation for a given *T*_int_, due to changes in the fraction of spin current that relaxes directly to the lattice via SOC instead of being absorbed by the magnetization to apply SOTs. These results uncover the critical role of spin relaxation rates of the magnetic layer and provide a unified understanding for the diverse SOT phenomena in ferromagnetic and antiferromagnetically-ordered systems.

## Results and discussion

### Sample details

**Fig. 1:**
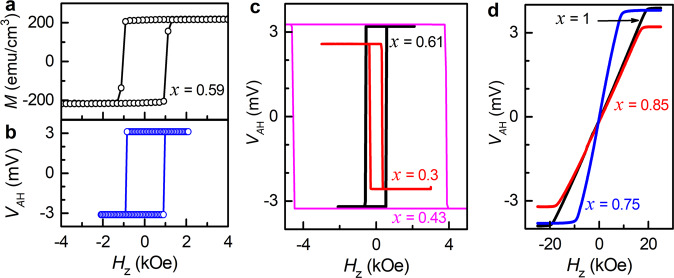
Magnetization and anomalous Hall voltage hysteresis. **a** Magnetization vs out-of-plane field (*H*_z_) and **b** Anomalous Hall voltage (*V*_AH_) vs *H*_z_ for Pt_0.75_Ti_0.25_ (5.6 nm)/Fe_0.59_Tb_0.41_ (8 nm) under a sinusoidal electric field *E* = 30 kV/m, indicating strong perpendicular magnetic anisotropy and a high coercivity of ≈1 kOe. *V*_AH_ vs *H*_z_ for Pt_0.75_Ti_0.25_ (5.6 nm)/Fe_*x*_Tb_1-*x*_ (8 nm) with **c** perpendicular (*x* = 0.3, 0.43, and 0.61) and **d** in-plane magnetic anisotropy (*x* = 0.75, 0.85, and 1).

For this work, we sputter-deposited Pt_0.75_Ti_0.25_ (5.6 nm)/Fe_*x*_Tb_1-*x*_ (8 nm) bilayers with different Fe volumetric concentrations (*x* = 0.3–1). The Pt_0.75_Ti_0.25_ layer, a dirty-limit Pt alloy with strong intrinsic spin Hall effect^17^, sources spin current that exerts SOT on the FIM Fe_*x*_Tb_1-*x*_. Each sample was deposited by co-sputtering on an oxidized Si substrate with a 1 nm Ta seed layer, and protected by a 2 nm MgO and a 1.5 nm Ta layer that was oxidized upon exposure to atmosphere. For electrical measurements, the samples were patterned into 5 × 60 µm^2^ Hall bars by photolithography and ion milling with a water-cooled stage. After processing, the magnetization hysteresis of the Fe_*x*_Tb_1-*x*_ measured from the anomalous Hall voltage (*V*_AH_) in patterned Hall bars shows fairly close coercivity (perpendicular depinning field) and squareness as the magnetization of unpatterned regions of the films measured by a superconducting quantum interference device (see Fig. 1a, b and Method). As shown in Fig. 1a–d, the Fe_*x*_Tb_1-*x*_ has strong bulk perpendicular magnetic anisotropy (PMA) for 0.3 ≤ *x* ≤ 0.62 and well-defined in-plane magnetic anisotropy for 0.75 ≤ *x* ≤ 1. All the PMA samples have large anisotropy fields (14.4–72.2 kOe, as estimated from the fits in Supplementary Fig. S2a) and square hysteresis loops for both the out-of-plane magnetization and anomalous Hall voltage.

### Strong variation of spin-orbit torques with composition and temperature

We performed harmonic Hall voltage response (HHVR) measurements^28,29^ by carefully separating out any potential thermoelectric effects (see Supplementary Note 2 for details). We choose the HHVR technique with excitation of a sinusoidal electric field *E* (typically 30 kV/m) because it allows very accurate, consistent determination of SOTs for both IMA and PMA samples^30–32^ without introducing significant thermal heating (Fig. 1a, b and Supplementary Fig. S4). We calculate the SOT efficiency using ${\xi }_{{{{{{\rm{DL}}}}}}}^{j}$ = (2*e/*$\hslash$)*M*_s_ *t*_FeTb_ *H*_DL_/*j*_c_^18^, where *e* is the elementary charge, $\hslash$ the reduced Plank’s constant, *t*_FeTb_ the Fe_*x*_Tb_1-*x*_ thickness, *M*_s_ the saturation magnetization of the Fe_*x*_Tb_1-*x*_ (Supplementary Note 1), and *j*_c_ = *E*/*ρ*_*xx*_ is the sinusoidal current density in the Pt_0.75_Ti_0.25_ with resistivity *ρ*_*xx*_ (*j*_c_ ≈ 2.2 × 10^6^A/cm^2^ for *E* = 30 kV/m). *H*_DL_ is the current-driven damping-like SOT field. The “planar Hall correction” is negligible for these PMA Fe_*x*_Tb_1-*x*_ samples (*V*_Ph_/*V*_AH_ < 0.1, Supplementary Fig. S3).

**Fig. 2:**
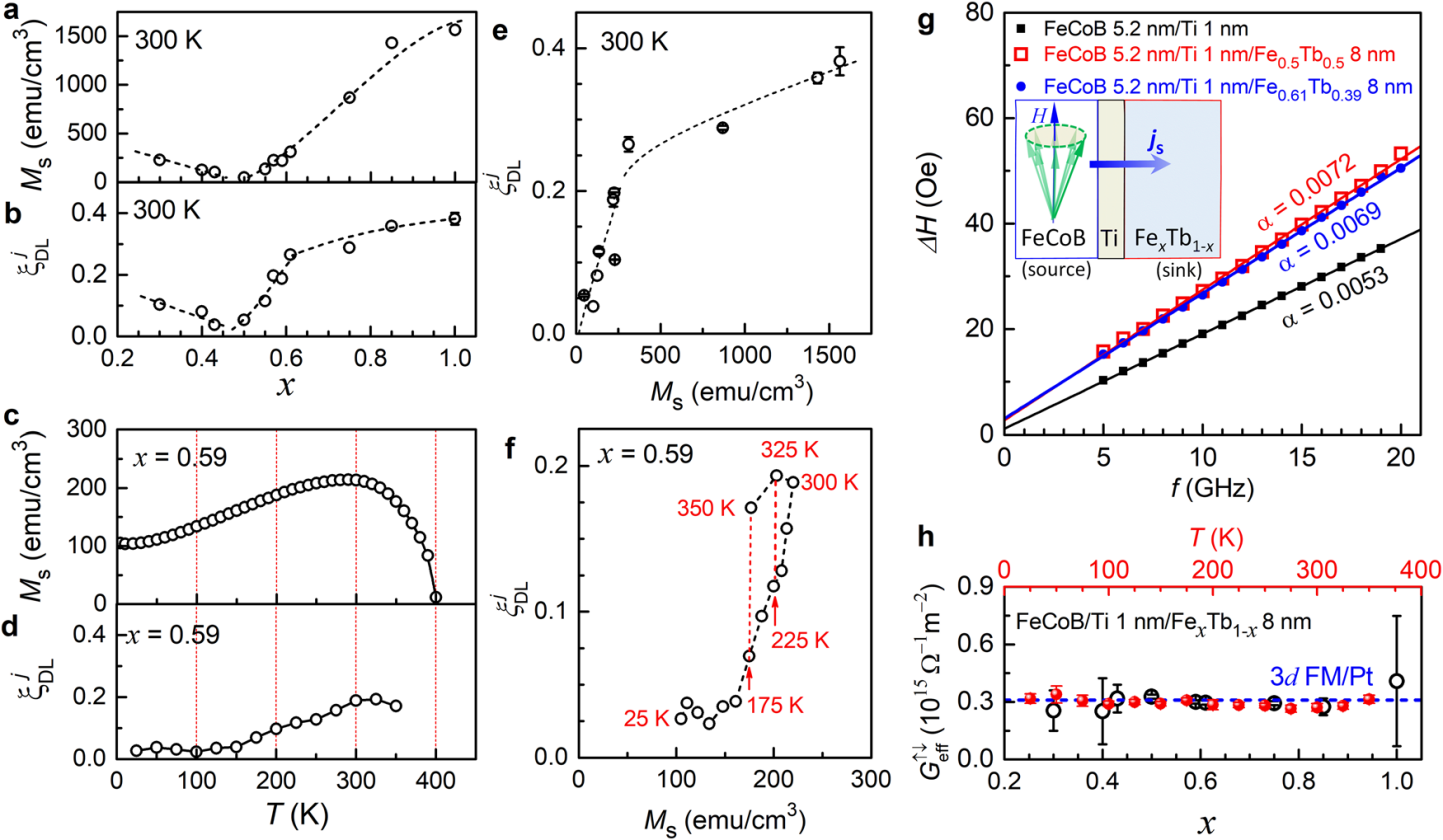
Spin-orbit toque and spin-mixing conductance. **a**, **b** Saturation magnetization (*M*_s_) and Damping-like SOT efficiency per unit current density (${\xi }_{{{{{{\rm{DL}}}}}}}^{j}$) for Pt_0.75_Ti_0.25_/Fe_*x*_Tb_1-*x*_ with different Fe concentration (*x*) at 300 K. **c**, **d** *M*_s_ and ${\xi }_{{{{{{\rm{DL}}}}}}}^{j}$ for the Pt_0.75_Ti_0.25_/Fe_0.59_Tb_0.41_ at different temperatures. **e** ${\xi }_{{{{{{\rm{DL}}}}}}}^{j}$ vs *M*_s_ for the Pt_0.75_Ti_0.25_/Fe_*x*_Tb_1-*x*_ with different Fe concentration (*x*) at 300 K. **f** ${\xi }_{{{{{{\rm{DL}}}}}}}^{j}$ vs *M*_s_ for the Pt_0.75_Ti_0.25_/Fe_0.59_Tb_0.41_ at different temperatures. **g** Frequency dependence of ferromagnetic resonance linewidth (*∆H*) of the FeCoB layer in FeCoB (5.2 nm)/Ti (1 nm), FeCoB (5.2 nm)/Ti (1 nm)/Fe_0.5_Tb_0.5_ (8 nm), and FeCoB (5.2 nm)/Ti (1 nm)/Fe_0.61_Tb_0.39_ (8 nm) samples. The solid lines represent linear fits, the slopes of which yield the damping. In (**a**–**e**) some error bars are smaller than the data points. **h** ${G}_{{{{{{\rm{eff}}}}}}}^{\uparrow \downarrow }$ of the FeCoB/Ti/Fe_*x*_Tb_1-*x*_ interfaces measured from spin pumping into the Fe_*x*_Tb_1-*x*_. The blue circles are for the composition series (300 K) and the red dots for the temperature series (*x* = 0.59). The blue dashed line represents ${G}_{{{{{{\rm{eff}}}}}}}^{\uparrow \downarrow }$ = 0.31 × 10^15^ Ω^−1^ m^−2^ previously reported for typical Pt/3*d* FM interfaces^33^. Error bars represent fitting uncertainty.

In Fig. 2a, b, we show the measured values of *M*_s_ and ${\xi }_{{{{{{\rm{DL}}}}}}}^{j}$ at 300 K for the Pt_0.75_Ti_0.25_/Fe_*x*_Tb_1-*x*_ bilayers with different Fe_*x*_Tb_1-*x*_ compositions (we refer to this as the composition series). *M*_s_ decreases monotonically by a factor of 33, from 1560 emu/cm^3^ for *x* = 1 (pure Fe, 3*d* FM) to 47 emu/cm^3^ for *x* = 0.5 (nearly full compensation), and then increases slowly as *x* further decreases. More details about the composition dependent magnetic properties are shown in Supplementary Note 1. As *x* decreases in the Fe-dominated regime (*x* ≥ 0.5), ${\xi }_{{{{{{\rm{DL}}}}}}}^{j}$ decreases by a factor of 7 at 300 K, first slowly from 0.38 ± 0.02 for *x* = 1 to 0.27 ± 0.01 for *x* = 0.61 and then more rapidly to 0.054 ± 0.002 for *x* = 0.5. ${\xi }_{{{{{{\rm{DL}}}}}}}^{j}$ increases slowly with decreasing *x* in the Tb-dominated regime (*x* < 0.5). The field-like SOT from the same HHVR measurements is smaller than ${\xi }_{{DL}}^{j}$ for each *x* and also varies with *x* (Supplementary Fig. S7). We also measured *M*_s_ and ${\xi }_{{{{{{\rm{DL}}}}}}}^{j}$ of Pt_0.75_Ti_0.25_/Fe_0.59_Tb_0.41_ as a function of temperature (we refer to this as the temperature series). Upon cooling from 350 K to 25 K, *M*_s_ and ${\xi }_{{DL}}^{j}$ for the Pt_0.75_Ti_0.25_/Fe_0.59_Tb_0.41_ sample are tuned by >2 times (Fig. 2c) and by >7.5 times (Fig. 2d), respectively. The dramatic tuning of ${\xi }_{{{{{{\rm{DL}}}}}}}^{j}$ by the Fe_*x*_Tb_1-*x*_ composition and temperature is a striking observation because it suggests a strong dependence of SOTs on some bulk properties of the Fe_*x*_Tb_1-*x*_, in contrast to ${\xi }_{{{{{{\rm{DL}}}}}}}^{j}$ for heavy metal (HM)/3*d* FM samples, which is insensitive to the type of the FM^33^ and temperature^34,35^. Apparently, ${\xi }_{{{{{{\rm{DL}}}}}}}^{j}$ for the Pt_0.75_Ti_0.25_/Fe_*x*_Tb_1-*x*_ correlates closely with net magnetization (Fig. 2a, b) but not in a proportional or monotonic manner (Fig. 2e, f), suggesting a rather critical role of the net magnetization as well as another bulk effect (which, as we discuss below, is spin-orbit scattering) in the determination of ${\xi }_{{{{{{\rm{DL}}}}}}}^{j}$.

### Robustness of the spin Hall ratio and the effective spin-mixing conductance

These strong variations cannot be explained by changes in either *θ*_SH_ or *T*_int_ (Eq. (1)). First, *θ*_SH_ is a property of the Pt_0.75_Ti_0.25_ layer, not the Fe_*x*_Tb_1-*x*_ layer. The Pt_0.75_Ti_0.25_ layer is made identically for all of the samples, and has a sufficiently large resistivity (*ρ*_*xx*_ = 135 µΩ cm) such that its properties can hardly be affected significantly by either a neighboring layer or temperature. We have verified that ${\xi }_{{{{{{\rm{DL}}}}}}}^{j}$ for a ferromagnetic Pt_0.75_Ti_0.25_ (5.6 nm)/FePt (8 nm) bilayer only has very weak temperature dependence (Supplementary Fig. S13), in good consistence with previous reports on other HM/3*d* FM samples^34,35^. We have also measured negligible SOT signal from the 8 nm Fe_*x*_Tb_1-*x*_ layers in control samples without a Pt_0.75_Ti_0.25_ layer (Supplementary Note 6), indicating that changes in our signals are not due to SOT arising from the Fe_*x*_Tb_1-*x*_ bulk. Note that a bulk torque of a magnetic layer is strongly thickness dependent^36,37^ and vanishes at small thicknesses of a few nm^36^.

As for the possibility of changes in *T*_int_, if we employ a drift-diffusion analysis^14–16^, the effect on *T*_int_ of spin backflow at the Pt_0.75_Ti_0.25_/Fe_*x*_Tb_1-*x*_ interface should be proportional to the effective spin-mixing conductance (${G}_{{{{{{\rm{eff}}}}}}}^{\uparrow \downarrow }$) of the interface, i.e., *T*_int_ ≈ 2${G}_{{{{{{\rm{eff}}}}}}}^{\uparrow \downarrow }$/*G*_PtTi_^38^, with *G*_PtTi_ = 1/*λ*_s_*ρ*_*xx*_ ≈ 1.3 × 10^5^ Ω^−1^ m^−1^ being the spin conductance^18,39^ of the Pt_0.75_Ti_0.25_. To quantify ${G}_{{{{{{\rm{eff}}}}}}}^{\uparrow \downarrow }$, we measure the change in the damping (*α*) of a precessing Fe_60_Co_20_B_20_ (= FeCoB) layer due to the absorption of the FeCoB-emitted spin current at the Fe_*x*_Tb_1-*x*_ interfaces (Fig. 2 g and Supplementary Fig. S8). The samples used here had the structure FeCoB (5.2 nm)/Ti (1 nm) and FeCoB (5.2 nm)/Ti (1 nm)/Fe_*x*_Tb_1-*x*_ (8 nm). Each of these samples is sputter-deposited on a 1 nm Ta seed layer and protected by capping with MgO (2 nm)/Ta (1 nm). The value of *α* for the FeCoB layers is determined from the linear dependence of the ferromagnetic resonance linewidth (*∆H*, half width at half maximum) on the frequency (*f*) using the relation *ΔH* = *ΔH*_0_ + 2π*αf/γ*, where *ΔH*_0_ is the inhomogeneous broadening of the linewidth and *γ* the gyromagnetic ratio. The damping enhancement of the FeCoB layer induced by spin pumping into the 8 nm Fe_*x*_Tb_1-*x*_ layers, ∆*α* = *α*_FeCoB/Ti/FeTb_ - *α*_FeCoB/Ti_, can be related to ${G}_{{{{{{\rm{eff}}}}}}}^{\uparrow \downarrow }$ as refs. ^40–42^

2 $$\triangle \alpha=\gamma {{{\hslash }}}^{2} {G}_{{{{{{\rm{eff}}}}}}}^{\uparrow \downarrow }/2{e}^{2}{M}_{{{{{{\rm{FeCoB}}}}}}}{t}_{{{{{{\rm{FeCoB}}}}}}}$$

where *t*_FeCoB_ = 5.2 nm and *M*_FeCoB_ = 1255 emu/cm^3^ is the saturation magnetization of the FeCoB layer as measured by SQUID. The value of *α* = 0.0053 for the bare FeCoB/Ti sample with no Fe_*x*_Tb_1-x_ coincides closely with the intrinsic damping of FeCoB (≈0.006^33^), indicating that the damping in this system does not contain any significant contributions from interfacial two-magnon scattering or spin memory loss. As shown in Fig. 2h, ${G}_{{{{{{\rm{eff}}}}}}}^{\uparrow \downarrow }$ of the FeCoB/Ti/Fe_*x*_Tb_1-*x*_ interfaces is insensitive to temperature and the Fe_*x*_Tb_1-*x*_ composition within experimental uncertainty, and has a value as high as that of typical 3*d* FM/Pt interfaces (≈0.31 × 10^15^ Ω^−1^ m^−2^) ^33^. This indicates that compensated Fe_*x*_Tb_1-*x*_ alloys act as spin sinks that are just as good as 3*d* FMs and Pt, and that there is no enhancement in the amount of spin reflection and backflow due to magnetic compensation. In principle, changes in *T*_int_ for SOT measurements could also arise from spin memory loss induced by interfacial SOC^29,43,44^, but this should be a minor effect for *T*_int_ of our un-annealed Pt_0.75_Ti_0.25_/Fe_*x*_Tb_1-*x*_ just as is the case of un-annealed Pt/Co^45^. As noted above, we also do not observe any enhancement in damping due to spin memory loss in the spin-pumping measurements. Note that the large and robust ${G}_{{{{{{\rm{eff}}}}}}}^{\uparrow \downarrow }$ for electron-mediated spin transport at the metallic Fe_*x*_Tb_1-*x*_ interface is in sharp contrast to that of ferrimagnetic insulator interfaces^46,47^ where thermal magnons mediate the spin transport such that a very low magnetic moment density reduces ${G}_{{{{{{\rm{eff}}}}}}}^{\uparrow \downarrow }$.

### Variation of SOT with the relative spin relaxation rates

**Fig. 3:**
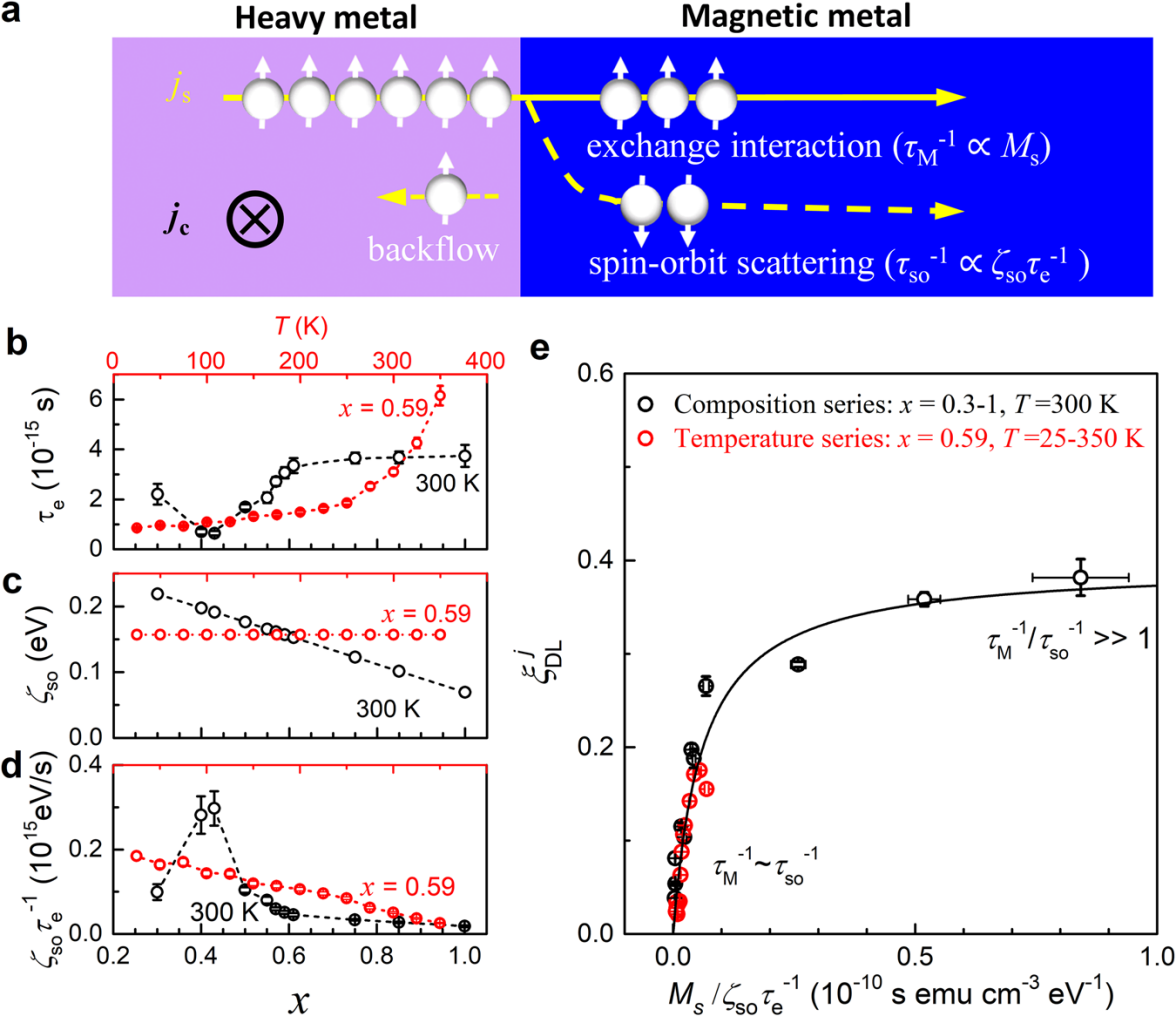
Variation of SOT with the relative spin relaxation rates. **a** Schematic of the spin relaxation processes that can influence the SOT, highlighting the competition between exchange interaction (with relaxation rate τ_M_^−1^ ∝ *M*_s_) and spin-orbit scattering (τ_so_^−1^ ∝ *ζ*_so_*τ*_e_^−1^). Only the spin current relaxed by exchange interaction contributes to SOTs. **b** Momentum scattering time (*τ*_e_), **c** Estimated SOC strength (*ζ*_so_), **d** *ζ*_so_*τ*_e_^−1^, and **e** ${\xi }_{{{{{{\rm{DL}}}}}}}^{j}$ of Pt_0.75_Ti_0.25_/Fe_*x*_Tb_1*-x*_ vs *M*_s_/*ζ*_so_*τ*_e_^−1^ for the composition series (*x* = 0.3–1, *T* = 300 K, black circles) and for the temperature series (*x* = 0.59, *T* = 25-300 K, red circles). The solid curve in (**e**) represents the fit of the data to Eq. (3). Error bars represent fitting uncertainty.

Since we have ruled out any significant change in *θ*_SH_ or *T*_int_ as contributing to the large variations we measure in ${\xi }_{{DL}}^{j}$ as a function of composition and temperature, these large variations must be due to physics that is not captured in the simple Eq. (1). We show below that spin relaxation induced by SOC in the bulk of the Fe_*x*_Tb_1-*x*_ layer is the most likely physics that is neglected in Eq. (1). As schematically shown in Fig. 3a, a spin current, in general, can be relaxed in a magnetic layer through two competing mechanisms: exchange-interaction-induced angular momentum transfer from spin current to magnetization (with a relaxation rate *τ*_M_^−1^ and a length scale of *λ*_dp_) and bulk spin-orbit-scattering-induced loss of spin angular momentum to the lattice (with a relaxation rate *τ*_so_^−1^ and a length scale of *λ*_s_). Theory^24–27^ and experiments^48,49^ have suggested that, in fully or partially compensated magnetic systems, the rate of spin angular momentum transfer via exchange interaction can be greatly decreased because of cancellations between exchange fields of antiferromagnetically-aligned magnetic sub-lattices, resulting in long *λ*_dp_ and low *τ*_M_^−1^. Spin-orbit scattering is well known to result in spin relaxation in both magnetic and non-magnetic materials^22,23^. While relatively weak in light, highly conductive 3*d* FMs (e.g., *λ*_s_ was measured to be 5–8 nm for Fe, Co, and CoFe at room temperature^22,23^), spin-orbit scattering becomes very strong in strong-SOC, resistive materials (e.g., dirty heavy metals^18^ and rare-earth FIMs) and substantially reduces *λ*_s_ and enhances *τ*_so_^−1^. This makes it possible for spin currents in FIMs to relax partially or even primarily via spin-orbit scattering to the lattice, instead of applying a spin-transfer torque to the magnetization.

We can consider how the SOT should scale as a function of the ratio *τ*_M_^−1^/*τ*_so_^−1^. Quantitative measurements of these rates (e.g., from the dependence on layer thicknesses of spin valve or spin-pumping experiments) are quite challenging because the bulk properties of Fe_*x*_Tb_1-*x*_^50^ and other ferrimagnetic alloys^48,49^ vary sensitively with the layer thicknesses^51–53^ (e.g., the magnetic compensation, the bulk PMA, the orientation of the magnetic easy axis, and resistivity all change with thickness). Nonetheless, it is reasonable to expect *τ*_M_^−1^ ∝ *M*_s_ for such ferrimagnetic alloys considering the canceling effects of the exchange fields from the antiferromagnetically-aligned magnetic sub-lattices. For the spin-orbit scattering rate, the Elliot-Yafet mechanism^51–53^ predicts *τ*_so_^−1^ ∝ *ζ*_so_*τ*_e_^−1^, where *ζ*_so_ is the bulk SOC strength and *τ*_e_^−1^ is the momentum scattering rate. One can thus expect *τ*_M_^−1^/*τ*_so_^−1^ = *kM*_s_*/ζ*_so_*τ*_e_^−1^, with *k* being a constant. Since only the spin current relaxed through exchange interaction with magnetization contributes to SOTs, we propose that

3 $${\xi }_{{{{{{\rm{DL}}}}}}}^{j}\approx {\xi }_{{{{{{\rm{DL}}}}}},0}^{j}{{\tau }_{{{{{{\rm{M}}}}}}}}^{-1}/\left({{\tau }_{{{{{{\rm{M}}}}}}}}^{-1}+{{\tau }_{{{{{{\rm{so}}}}}}}}^{-1}\right)={\xi }_{{{{{{\rm{DL}}}}}},0}^{j}{\left(1+{\left({{kM}}_{{{{{{\rm{s}}}}}}}/{\zeta }_{{{{{{\rm{so}}}}}}}{{\tau }_{{{{{{\rm{e}}}}}}}}^{-1}\right)}^{-1}\right)}^{-1}$$

where ${\xi }_{{{{{{\rm{DL}}}}}},0}^{j}$ is the value of ${\xi }_{{{{{{\rm{DL}}}}}}}^{j}$ in the limit *τ*_M_^−1^/*τ*_so_^−1^ ≫ 1.

Figure 3b–d shows the estimated values of *τ*_e_, *ζ*_so_, and *ζ*_so_*τ*_e_^−1^ for both our composition series (*x* = 0.3-1, *T* = 300 K, black symbols) and our temperature series (*x* = 0.59, *T* = 25–350 K, red symbols). Here, the value of *τ*_e_ is a rough estimate from the resistivity of the Fe_*x*_Tb_1-*x*_ following Drude model *ρ*_FeTb_ = m*/*ne*^2^*τ*_e_, where m* is the effective mass of the carriers and *n* is the carrier density measured from the ordinary Hall coefficient (*R*_OH_ = 1/*ne* for a single-band model^54,55^, see Supplementary Note 5 for more details). *τ*_e_ of the Fe_*x*_Tb_1-*x*_ varies by a factor of ≈6 by composition and a factor of ≈7 by temperature, suggesting a significant tuning of the Fermi surface properties. The increase of *τ*_e_ in the Fe_*x*_Tb_1-*x*_ metal with raising temperature likely originates from electron-electron interaction^56^ (*ρ*_FeTb_ $\propto$ *T* ^1/2^, see Supplementary Fig. S14a) or magnetic Brillouin zone scattering (the periodic potentials due to antiferromagnetic alignment of the magnetic sublattices can produce an additional magnetic Brillouin zone, of smaller volume in *k*-space than the ordinary lattice potential, whose planes further incise and contort the Fermi surface^57^). Both electron-electron interaction and magnetic Brillouin zone lead to additional electron scattering manifesting as a resistivity upturn upon cooling.^56,57^ The average SOC strength of the Fe_*x*_Tb_1-*x*_ is estimated as *ζ*_so_ ≈ *xζ*_so,Fe_ + (1-*x*)*ζ*_so,Tb_ following the linear dependence on alloy composition of the bulk SOC^58^ and the theoretical values^59^ of *ζ*_so,Fe_ = 0.069 eV for Fe and *ζ*_so,Tb_ = 0.283 eV for Tb (while the actual values of *ζ*_so,Fe_ and *ζ*_so,Tb_ within the amorphous Fe_*x*_Tb_1-*x*_, which are not trivial to obtain, may be slightly different from the theoretical ones, this estimation should, at least, provide a reasonable functional approximation for the expected dramatic variation of *ζ*_so_ as a function of the Fe_*x*_Tb_1-*x*_ composition, from light Fe to Tb-rich Fe_0.3_Tb_0.7_). We estimate that *ζ*_so_*τ*_e_^−1^ decreases by a factor of >16 as *x* varies between 0.3 to 1 and by a factor of >7 as temperature increases from 25 K to 350 K (Fig. 3d). In Fig. 3e, we plot ${\xi }_{{DL}}^{j}$ as a function of *M*_s_*/ζ*_so_*τ*_e_^−1^ for both the composition series and the temperature series. As *M*_s_*/ζ*_so_*τ*_e_^−1^, or equivalently *τ*_M_^−1^/*τ*_so_^−1^, decreases, we find that ${\xi }_{{DL}}^{j}$ decreases first slowly and then rapidly towards a vanishing value. The variation of ${\xi }_{{DL}}^{j}$ with *M*_s_*/ζ*_so_*τ*_e_^−1^ can be fit very well by Eq. (3) with ${\xi }_{{{{{{\rm{DL}}}}}},0}^{j}$ = 0.395 ± 0.022 and *k* = (1.66 ± 0.23) × 10^11 ^s^−1^ emu^−1^ cm^3^ eV. Note that the applicability of the Drude model, the approximated value of m*, and the single-band model for the ordinary Hall effect for estimating *τ*_e_^−1^ is not essential for our conclusion of the variation of ${\xi }_{{DL}}^{j}$with relative spin relaxation rates, since similar scaling in Fig. 3e is present even when simply plotting ${\xi }_{{DL}}^{j}$ as a function of *M*_s_*/ζ*_so_*ρ*_*xx*_ (Supplementary Fig. S11). In the above discussions, we ignored any effect of local distribution of magnetizations (also known as sperimagnetism^50^) because it, if present, may only indirectly affect the average spin relaxation rates of spin-magnetization exchange interaction and spin-orbit scattering via reducing the net magnetization and strengthening SOC-related momentum scattering of spin carriers, respectively.

Since the strong variation of ${\xi }_{{DL}}^{j}$with relative spin relaxation rates we propose here is unlikely to be specific just to the HM/Fe_*x*_Tb_1-*x*_ system (as indicated by the widespread presence of spin-orbit scattering in various materials^22,23^ and by the general fact that the SOT provided by a given spin-current generator is significantly weaker on FIMs than on FMs, see Table 1), we generalize Eq. (1) as

4 $${\xi }_{{DL}}^{j}={T}_{{{{{{\rm{int}}}}}}}{\theta }_{{{{{{\rm{SH}}}}}}}{{\tau }_{{{{{{\rm{M}}}}}}}}^{-1}/\left({{\tau }_{{{{{{\rm{M}}}}}}}}^{-1}+{{\tau }_{{{{{{\rm{so}}}}}}}}^{-1}\right).$$

**Table 1:** Comparison of ${\xi }_{{{{{{\rm{DL}}}}}}}^{{{{{{\rm{j}}}}}}}$ for FIMs and 3*d* FMs in contact with spin current sources that have similar resistivities, thicknesses, and thus similar values of *θ*_SH_ and *T*_int_

spin current source	${\xi }_{{{{{{\rm{DL}}}}}}}^{{{{{{\rm{j}}}}}}}$		
	FIM	3*d* FM	ratio
Ta	−0.03 (CoTb)^5^	−0.12 (FeCoB)^10^	4
W	−0.04 (CoTb)^67^	−0.44 (FeCoB)^68^	11
Pt	0.017 (CoTb)^5^	0.15 (Co, FeCoB)^10^	8.8
Pt/NiO	0.09 (CoTb)^6^	0.6 (FeCoB)^35^	6.7
Pt_0.75_Ti_0.25_	0.05 (Fe_0.5_Tb_0.5_)	0.38 (Fe)	7.6
Bi_2_Se_3_	0.13 (GdFeCo)^7^	3.5 (NiFe)^69^	27

This generalized equation should apply to FMs, FIMs, and AFs that are metals or insulators. In magnetic insulators, an incident spin current carried by magnons transfers angular momentum to the magnetization via exchange interaction (with spin relaxation rate *τ*_M_^−1^) and also to the lattice via spin-orbit scattering of spin carriers (with spin relaxation rate *τ*_so_^−1^).

We note that our conclusions are contrary to some previous experiments, which reported ${\xi }_{{{{{{\rm{DL}}}}}}}^{j}$ to remain constant^60–62^ or even diverge^63^ near the magnetic compensation point of HM/CoTb or HM/CoFeGd bilayers. While it might be possible that *τ*_M_^−1^ /*τ*_so_^−1^ is different in CoTb and CoFeGd compared to Fe_*x*_Tb_1-*x*_ (e.g., Gd has zero atomic orbital angular momentum^8^ and thus considerably weaker SOC than Tb), we also question these previous conclusions for a variety of technical experimental reasons. In three of the previous experiments, the PMA of the FIM layer was weak and showed gradual magnetization hysteresis^62^, non-parabolic first-harmonic signal in HHVR measurements^60,62,63^, and/or non-linear second-harmonic signal in HHVR measurements^60,62^ as a function of a small in-plane applied magnetic field. This indicates magnetization behavior outside of the simple macrospin model assumed in the HHVR analysis. The HHVR results in refs. ^62,63^ also applied “planar Hall correction”, the latter, if significant, causes erroneous estimates of ${\xi }_{{DL}}^{j}$ (see refs. ^30,64–66^ for more discussions). References ^60,61^ reported substantial changes of sample properties before and after device patterning, resulting in large uncertainties in the estimation of *M*_s_ and ${\xi }_{{DL}}^{j}$for those samples. The loop-shift measurements in ref. ^61^ also applied large *dc* current densities of ~10^7 ^A/cm^2^, close to the switching current density, in the resistive Ta/Co_*x*_Tb_1-*x*_ samples, leading to considerable Joule heating that had significantly altered the temperature, *M*_s_, and ferrimagnetic compensation points of those samples. The latter resulted in additional uncertainties in those loop-shift results of ${\xi }_{{DL}}^{j}$ and ultimately prevented resolving the variation of ${\xi }_{{DL}}^{j}$ with the bulk properties of FIMs (i.e., *τ*_M_^−1^ /*τ*_so_^−1^) in that work.

### Scientific implications

**Fig. 4:**
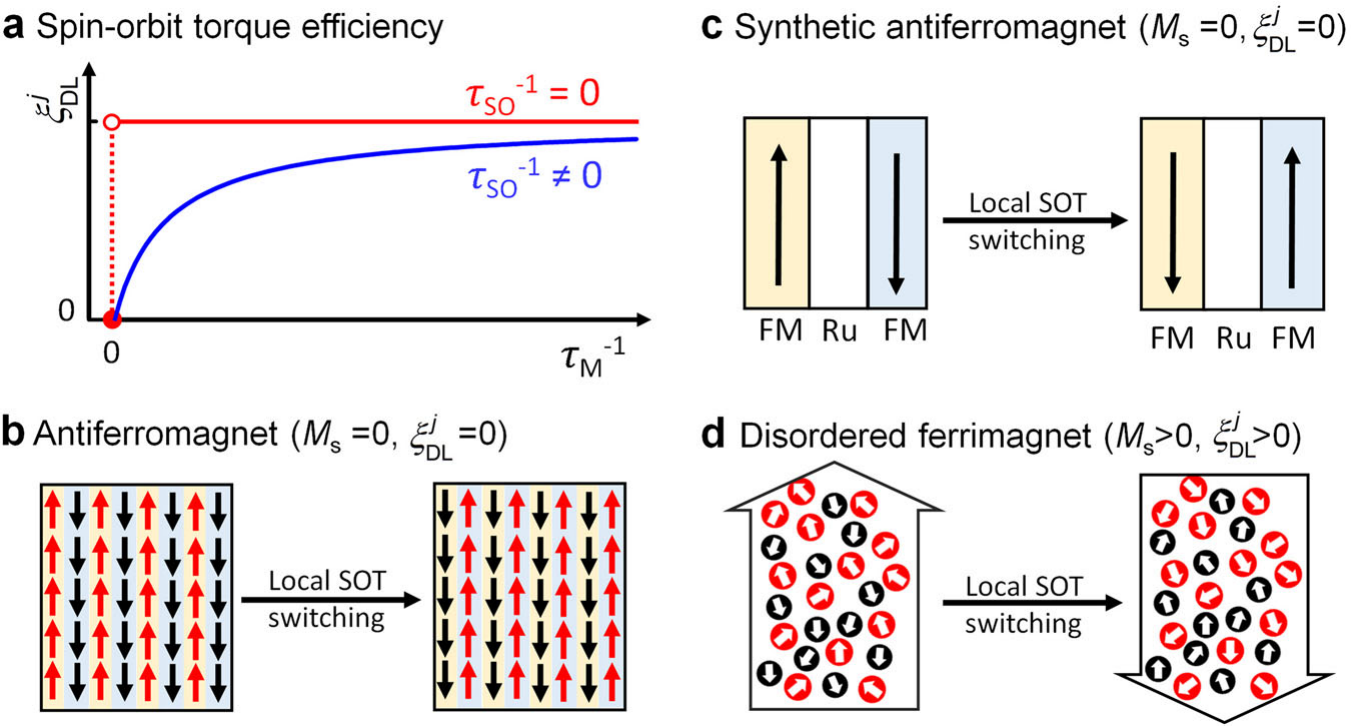
Schematic depicts of the implications. **a** Dependence on *τ*_M_^−1^ (∝ *M*_s_) of the efficiency of the damping-like spin-orbit torque (${\xi }_{{{{{{\rm{DL}}}}}}}^{j}$) exerted by a given spin current on a magnetic layer with zero spin-orbit scattering (*τ*_so_^−1^ = 0, red) and non-negligible spin-orbit scattering (*τ*_so_^−1^ ≠ 0, blue), highlighting the critical role of spin-orbit scattering. “Potential” spin current switching of local atomic magnetizations by *local* spin-orbit torque in **b** perfectly compensated single-layer antiferromagnet, **c** perfectly compensated synthetic antiferromagnet, and **d** disordered ferrimagnet, with negligible barrier against switching (magnetic anisotropy, pinning, damping, etc.). In the case of perfectly compensated antiferromagnets, a spin current generates zero net spin-orbit torque and zero net magnetization change.

Our finding of the critical role of the relative rates of spin-orbit-induced relaxation to the lattice versus spin transfer to the magnetization has important implications for this field and resolves outstanding puzzles in previous experiments. We first schematically demonstrate in Fig.4a the effect of spin-orbit scattering on SOTs suggested by Eq. (4). Only in absence of spin-orbit scattering (*τ*_so_^−1^ = 0), should the simple form of Eq. (1) apply such that ${\xi }_{{DL}}^{j}$ is independent of *τ*_M_^−1^ and thus *M*_s_ for a sufficiently thick magnetic layer with nonzero *M*_s_. This is a good approximation only for magnetic materials with *τ*_M_^−1^ /*τ*_so_^−1^ ≫ 1, e.g., 3*d* FMs that have high *M*_s_, low resistivity, and weak SOC. However, in the presence of non-negligible spin-orbit scattering (*τ*_so_^−1^ > 0), ${\xi }_{{DL}}^{j}$decreases more and more rapidly with reducing *τ*_M_^−1^/*τ*_so_^−1^ (with *τ*_M_^−1^ ∝ *M*_s_). This is generally the case of uncompensated “AF” domains (*M*_s_ > 0 emu/cm^3^) and FIMs with strong SOC and large resistivities (e.g., Fe_*x*_Tb_1-*x*_ and Co_*x*_Tb_1-*x*_). However, ${\xi }_{{DL}}^{j}$ always diminishes at zero *τ*_M_^−1^ (*M*_s_ = 0 emu/cm^3^), e.g., in perfectly compensated FIMs and AFMs and non-magnetic materials.

Therefore, SOTs will be reduced in any experiments which use magnetic free layers for which *τ*_M_^−1^ is not much greater than *τ*_so_^−1^ or not sufficiently large to allow complete spin relaxation within the layer thickness. The condition *τ*_M_^−1^/ *τ*_so_^−1^ ≪ 1 resolves the puzzles why the measured ${\xi }_{{DL}}^{j}$values for SOT acting on nearly-compensated FIMs are often several to over 20 times smaller than for corresponding measurements using 3*d* FMs (see Table 1 for a few representative examples with spin current sources that have similar resistivities, thicknesses, and thus similar values of *θ*_SH_ and *T*_int_)^5–7,10,35,67–69^. We have also verified from study of a large number of samples that *τ*_M_^−1^/(*τ*_M_^−1^ + *τ*_so_^−1^) is 0.58 for 5 nm Co_0.65_Tb_0.35_ layers at room temperature such that ${\xi }_{{{{{{\rm{DL}}}}}}}^{j}$ for Pt-X/Co_0.65_Tb_0.35_ is only 58% of that of Pt-X/Co for given Pt-X (Pt-X being Pt-based alloys and multilayers)^70^. Spin-orbit scattering within the magnetic layer should, therefore, be minimized and the average exchange coupling maximized for efficient SOT devices.

Taking into account the relative spin relaxation rates also clarifies the ongoing debate as to whether AFs can be switched at all by SOTs^11–13^. The previous observation of current-driven switching of uncompensated magnetic domains or magnetizations embedded within AF hosts using transport or imaging methods^71–75^ (which is not real switching of AF Néel vector) is naturally explained by the small but sizable SOTs in nearly but not fully-compensated systems, while the absence of macroscopic transport evidence of current switching of some more uniform HM/AF^11–13^ is well consistent with the diminishment of SOTs in fully-compensated systems (Fig. 4a).

While in principle a spin current still has the potential to switch the local nonzero atomic magnetizations within a perfectly compensated FIMs, single-layer AFs, and synthetic AFs (e.g., FM/Ru/FM) by *local* SOT on each magnetic site (Fig. 4b–d), such switching is unfortunately very inefficient in overcoming the switching barriers of the samples (e.g., magnetic anisotropy, pinning field, damping, etc.). It is rather typical that the effective magnetic anisotropy fields and pinning fields (i.e., the coercivities in the magnetization hysteresis) of hard rare-earth transition-metal FIMs^50,60–62^ and synthetic AFs^11^ turn to diverge at the magnetization compensation point, making them unswitchable for a realistic spin current. Collective 180^o^ reversal of the Néel vector of collinear AFs also requires net SOT to overcome the barriers against switching (e.g., magnetic anisotropy, pinning, damping, etc.), which is challenging to achieve, particularly so in present of spin-orbit scattering. Therefore, fully-compensated FIMs and AFs themselves are most likely not an option as “Néel-vector-type” free layer of magnetic memory in terms of electrical writability and readability. We also note that field-like effective SOT field is too weak to reach the spin-flop field required for 90^o^ rotation of Néel vector (typically of the order of 1−10^3 ^kOe)^76^.

In summary, we have shown that the strength of SOTs depends critically on the ratio of rate of spin-orbit-induced spin relaxation within a magnetic layer relative to the rate of exchange-induced spin transfer to the magnetization. We find experimentally that SOT efficiencies decrease strongly upon approaching the magnetic compensation point in ferrimagnetic Fe_*x*_Tb_1-*x*_ due to a decrease in the rate of exchange-induced spin transfer on account of partial cancellation between the oppositely-directed exchange interactions from the magnetic sub-lattices. Near the compensation point, spin-orbit-induced spin relaxation dominates over spin transfer to the magnetization so that the measured SOT goes to zero. These results suggest the breakdown of the “interfacial torques” concept in FIMs and AFs. We find no indication of any dependence of the spin transparency of Fe_*x*_Tb_1-*x*_ interfaces on the degree of compensation. Our finding suggests that it will be essential to modify spin transport models that assumed an infinite *τ*_M_^−1^ to include spin decoherence by spin-orbit scatting on an equal footing with dephasing by the exchange interaction. This work provides not only a unified understanding of the very different efficiencies of SOTs that have been reported in the literature for FMs, FIMs, and AFs, but also insight about how the different sources of spin relaxation should be optimized in the design of FIMs and AFs for spintronic technologies^8,9^.

## Methods

### Sample fabrication

**Fig. 5:**
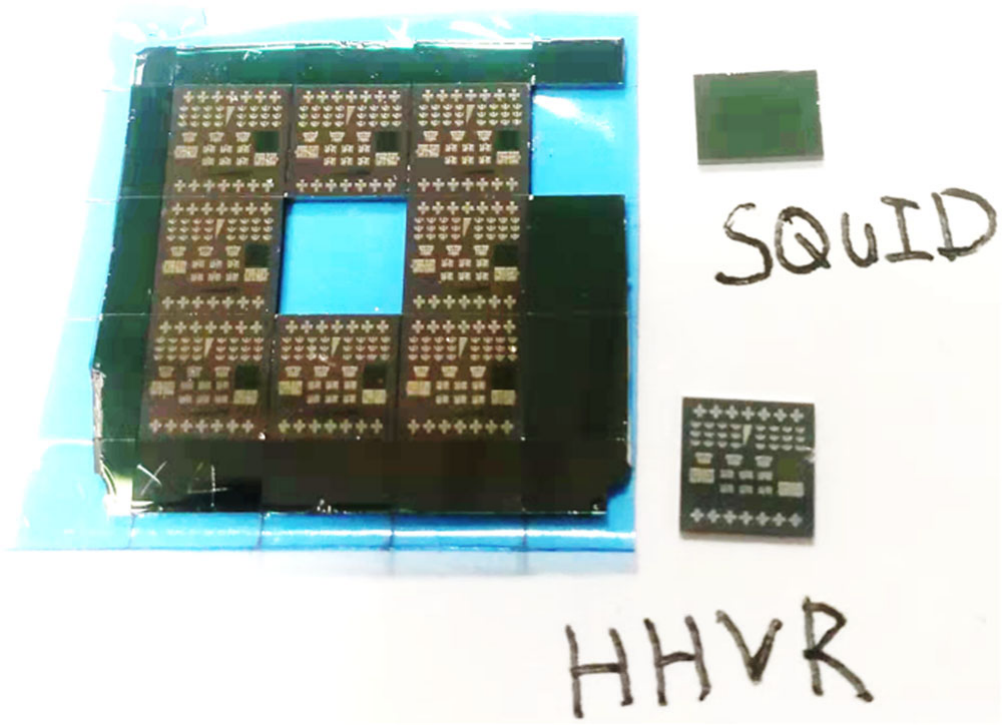
Photo of a Pt_0.75_Ti_0.25_/Fe_*x*_Tb_1-*x*_ sample separated into pieces by a dicing saw. The patterned pieces are used for HHVR measurements and un-patterned regions for SQUID measurements. The “SQUID” pieces underwent the same processing as the Hall bars during the device fabrication, providing good consistency between the electrical and magnetic properties of the samples in our analyses.

Samples for this study includes Pt_0.75_Ti_0.25_ (5.6 nm)/Fe_*x*_Tb_1-*x*_ (8 nm) bilayers, FeCoB (5.2 nm)/Ti (1 nm)/Fe_*x*_Tb_1-*x*_ (8 nm), FeCoB (5.2 nm)/Ti (1 nm), Pt_0.75_Ti_0.25_ (5.6 nm)/FePt (8 nm), and Fe_*x*_Tb_1-*x*_ (8 nm), with x being the Fe volumetric concentration (*x* = 0.3−1). Each sample was sputter-deposited on an oxidized Si substrate with a 1-nm Ta seed layer, and protected by a 2 nm MgO and a 1.5 nm Ta layer that was oxidized upon exposure to atmosphere. The FeCoB (5.2 nm)/Ti (1 nm)/Fe_*x*_Tb_1-*x*_ (8 nm) and FeCoB (5.2 nm)/Ti (1 nm) samples were diced into pieces for ferromagnetic resonance measurements. The other samples (2 × 2 cm in area) were partly patterned into 5 × 60 µm^2^ Hall bars by photolithography and ion milling with a water-cooled stage. After processing, the samples were separated into pieces by a dicing saw (Fig. 5). The patterned pieces were used for harmonic Hall voltage response (HHVR) measurements, and un-patterned regions of the films for magnetization characterizations using a superconducting quantum interference device (SQUID). The “SQUID” pieces underwent the same processing as the Hall bars during the device fabrications, providing good consistency between the electrical and magnetic properties of the samples in our analyses.

### Measurements

The saturation magnetization of each sample was measured by a Quantum Design SQUID as a function of magnetic field and temperature. The SOTs were measured using harmonic Hall voltage response (HHVR) measurements, during which a Signal Recovery DSP Lock-in Amplifier (Model 7625) or a Stanford Research System Lock-in Amplifier (Model SR830) was used to source a sinusoidal electric field *E* (typically 30 kV/m) onto the Hall bars and to detect the first and second harmonic Hall voltage responses. The magnetic damping was measured using flip-chip ferromagnetic resonance with a radio frequency signal generator, two Stanford Research System Lock-in Amplifiers (Model SR830), and a sweeping in-plane magnetic field.

### **Reporting summary**

Further information on research design is available in the Nature Portfolio Reporting Summary linked to this article.

## Supplementary information

Supplementary Information

Peer Review File

## Data Availability

Source data are provided with this paper.

## Source data

Source Data

## References

[1] Miron IM (2011). Perpendicular switching of a single ferromagnetic layer induced by in-plane current injection. Nature.

[2] Liu L (2012). Spin-torque switching with the giant spin Hall effect of tantalum. Science.

[3] Caretta L (2018). Fast current-driven domain walls and small skyrmions in a compensated ferrimagnet. Nat. Nanotechnol..

[4] Cai K (2020). Ultrafast and energy-efficient spin–orbit torque switching in compensated ferrimagnets. Nat. Electron..

[5] Han J (2017). Room-temperature spin-orbit torque switching induced by a topological insulator. Phys. Rev. Lett..

[6] Wang H (2019). Spin-orbit-torque switching mediated by an antiferromagnetic insulator. Phys. Rev. Applied.

[7] Wu H (2019). Spin-orbit torque switching of a nearly compensated ferrimagnet by topological surface states. Adv. Mater..

[8] Radu, F. & Sánchez-Barriga, J. Ferrimagnetic heterostructures for applications in magnetic recording. *Novel Magnetic Nanostructures* 267–331 (Elsevier, 2018).

[9] Zhang Z (2021). 3D ferrimagnetic device for multi-bit storage and efficient in-memory computing. IEEE Electron. Device Lett..

[10] Pai C-F, Mann M, Tan A J, Beach G S D (2016). Determination of spin torque efficiencies in heterostructures with perpendicular magnetic anisotropy. Phys. Rev. B.

[11] Ma Q (2020). Spin orbit torque switching of synthetic Co/Ir/Co trilayers with perpendicular anisotropy and tunable interlayer coupling. Appl. Phys. Lett..

[12] Chiang CC, Huang SY, Qu D, Wu PH, Chien CL (2019). Absence of evidence of electrical switching of the antiferromagnetic Néel vector. Phys. Rev. Lett..

[13] Zhang P, Finley J, Safi T, Liu L (2019). Quantitative study on current-induced effect in an antiferromagnet insulator/Pt bilayer film. Phys. Rev. Lett..

[14] Haney PM, Lee HW, Lee KJ, Manchon A, Stiles MD (2013). Current induced torques and interfacial spin-orbit coupling: Semiclassical modeling. Phys. Rev. B.

[15] Chen Y-T (2013). Theory of spin Hall magnetoresistance. Phys. Rev. B.

[16] Amin VP, Stiles MD (2016). Spin transport at interfaces with spin-orbit coupling: phenomenology. Phys. Rev. B.

[17] Zhu L, Ralph DC, Buhrman RA (2021). Maximizing spin-orbit torque generated by the spin Hall effect of Pt. Appl. Phys. Rev..

[18] Nguyen M-H, Ralph D C, Buhrman R A (2016). Spin torque study of the spin Hall conductivity and spin diffusion length in platinum thin films with varying resistivity. Phys. Rev. Lett..

[19] Stiles MD, Zangwill A (2002). Anatomy of spin-transfer torque. Phys. Rev. B.

[20] Ghosh A, Auffret S, Ebels U, Bailey WE (2012). Penetration depth of transverse spin current in ultrathin ferromagnets. Phys. Rev. Lett..

[21] Taniguchi T, Yakata S, Imamura H, Ando Y (2008). Penetration depth of transverse spin current in ferromagnetic metals. IEEE Trans. Magn..

[22] Bass J, Pratt WP (2007). Spin-diffusion lengths in metals and alloys, and spin-flipping at metal/metal interfaces: An experimentalist’s critical review. J. Phys.: Condens. Matter.

[23] Zahnd G (2018). Spin diffusion length and polarization of ferromagnetic metals measured by the spin-absorption technique in lateral spin valves. Phys. Rev. B.

[24] Núñez AS, Duine RA, Haney P, MacDonald AH (2006). Theory of spin torques and giant magnetoresistance in antiferromagnetic metals. Phys. Rev. B.

[25] Haney PM (2007). Ab initio giant magnetoresistance and current-induced torques in Cr/Au/Cr multilayers. Phys. Rev. B.

[26] Xu Y, Wang S, Xia K (2008). Spin-transfer torques in antiferromagnetic metals from first principles. Phys. Rev. Lett..

[27] Baltz V (2018). Antiferromagnetic spintronics. Rev. Mod. Phys..

[28] Avci CO (2014). Interplay of spin-orbit torque and thermoelectric effects in ferromagnet/normal-metal bilayers. Phys. Rev. B.

[29] Zhu L, Ralph DC, Buhrman RA (2019). Spin-orbit torques in heavy-metal–ferromagnet bilayers with varying strengths of interfacial spin-orbit coupling. Phys. Rev. Lett..

[30] Zhu LJ (2019). Strong damping-like spin-orbit torque and tunable Dzyaloshinskii–Moriya interaction generated by low-resistivity Pd_1−*x*_Pt_*x*_ alloys. Adv. Funct. Mater..

[31] Zhu L, Ralph DC, Buhrman RA (2018). Highly efficient spin-current generation by the spin Hall effect in Au_1−*x*_ Pt_*x*_. Phys. Rev. Appl..

[32] Zhu L (2019). Enhancing spin-orbit torque by strong interfacial scattering from ultrathin insertion layers. Phys. Rev. Appl..

[33] Zhu L, Ralph DC, Buhrman RA (2019). Effective spin-mixing conductance of heavy-metal–ferromagnet interfaces. Phys. Rev. Lett..

[34] Ou Y, Pai C-F, Shi S, Ralph DC, Buhrman RA (2016). Origin of fieldlike spin-orbit torques in heavy metal/ferromagnet/oxide thin film heterostructures. Phys. Rev. B.

[35] Zhu L, Zhu L, Buhrman RA (2021). Fully spin-transparent magnetic interfaces enabled by the insertion of a thin paramagnetic NiO layer. Phys. Rev. Lett..

[36] Zhu L, Zhang XS, Muller DA, Ralph DC, Buhrman RA (2020). Observation of strong bulk damping-like spin-orbit torque in chemically disordered ferromagnetic single layers. Adv. Funct. Mater..

[37] Liu Q, Zhu L, Zhang XS, Muller DA, Ralph DC (2022). Giant bulk spin–orbit torque and efficient electrical switching in single ferrimagnetic FeTb layers with strong perpendicular magnetic anisotropy. Appl. Phys. Rev..

[38] Pai C-F, Ou Y, Vilela-Leao LH, Ralph DC, Buhrman RA (2015). Dependence of the efficiency of spin Hall torque on the transparency of Pt/ferromagnetic layer interfaces. Phys. Rev. B.

[39] Sagasta E (2016). Tuning the spin Hall effect of Pt from the moderately dirty to the superclean regime. Phys. Rev. B.

[40] Mosendz O (2010). Quantifying spin Hall angles from spin pumping: experiments and theory. Phys. Rev. Lett..

[41] Czeschka FD (2011). Scaling behavior of the spin pumping effect in ferromagnet-platinum bilayers. Phys. Rev. Lett..

[42] Heinrich B (2011). Spin pumping at the magnetic insulator (YIG)/normal metal (Au) interfaces. Phys. Rev. Lett..

[43] Liu Y, Yuan Z, Wesselink RJH, Starikov AA, Kelly PJ (2014). Interface enhancement of gilbert damping from first principles. Phys. Rev. Lett..

[44] Rojas-Sánchez J-C (2014). Spin pumping and inverse spin Hall effect in platinum: the essential role of spin-memory loss at metallic interfaces. Phys. Rev. Lett..

[45] Zhu L, Buhrman RA (2019). Maximizing spin-orbit-torque efficiency of Pt/Ti multilayers: trade-off between intrinsic spin Hall conductivity and carrier lifetime. Phys. Rev. Appl..

[46] Jia X, Liu K, Xia K, Bauer GEW (2011). Spin transfer torque on ferrimagnetic insulators. EPL.

[47] Shao Q (2018). Role of dimensional crossover on spin-orbit torque efficiency in magnetic insulator thin films. Nat. Commun..

[48] Yu J (2019). Long spin coherence length and bulk-like spin–orbit torque in ferrimagnetic multilayers. Nat. Mater..

[49] Lim Y (2021). Dephasing of transverse spin current in ferrimagnetic alloys. Phys. Rev. B.

[50] Hebler B, Hassdenteufel A, Reinhardt P, Karl H, Albrecht M (2016). Ferrimagnetic Tb–Fe Alloy thin films: composition and thickness dependence of magnetic properties and all-optical switching. Front Mater..

[51] Elliott RJ (1954). Theory of the effect of spin-orbit coupling on magnetic resonance in some semiconductors. Phys. Rev..

[52] Yafet Y (1963). g Factors and spin-lattice relaxation of conduction electrons. Solid State Phys..

[53] Boguslawski P (1980). Electron-electron spin-flip scattering and spin relaxation in III–V and II–VI semiconductors. Solid State Commun..

[54] Cottam MG, Stinchcombe RB (1968). The theory of the ordinary Hall coefficient of iron at low temperatures. J. Phys. C.

[55] Rhyne JJ (1969). Anomalous and ordinary Hall effect in terbium. J. Appl. Phys..

[56] Zhu L, Zhao JH (2017). Anomalous resistivity upturn in epitaxial L2_1_-Co_2_MnAl films. Sci. Rep..

[57] Meaden GT (1971). Conduction electron scattering and the resistance of the magnetic elements. Contemp. Phys..

[58] Kim D, Liu F (2022). Topological alloy engineering and locally linearized gap dependence on concentration. Phys. Rev. B.

[59] Shanavas KV, Popović ZS, Satpathy S (2014). Theoretical model for Rashba spin-orbit interaction in *d* electrons. Phys. Rev. B.

[60] Ham WS (2017). Temperature dependence of spin-orbit effective fields in Pt/GdFeCo bilayers. Appl. Phys. Lett..

[61] Finley J, Liu L (2016). Spin-orbit-torque efficiency in compensated ferrimagnetic cobalt-terbium alloys. Phys. Rev. Applied.

[62] Ueda K, Mann M, de Brouwer PWP, Bono D, Beach GSD (2017). Temperature dependence of spin-orbit torques across the magnetic compensation point in a ferrimagnetic TbCo alloy film. Phys. Rev. B.

[63] Mishra R (2017). Anomalous current-induced spin torques in ferrimagnets near compensation. Phys. Rev. Lett..

[64] Torrejon J (2014). Interface control of the magnetic chirality in CoFeB/MgO heterostructures with heavy-metal underlayers. Nat. Commun..

[65] Karimeddiny S, Cham TM, Ralph DC, Luo YK (2021). Sagnac interferometry for high-sensitivity optical measurements of spin-orbit torque. arXiv.

[66] Zhu L, Li J, Zhu L, Xie X (2022). Boosting spin-orbit-torque efficiency in spin-current-generator/magnet/oxide superlattices. Phys. Rev. Appl..

[67] Peng C-W, Liao W-B, Chen T-Y, Pai C-F (2021). Efficient spin-orbit torque generation in semiconducting WTe_2_ with hopping transport. ACS Appl. Mater. Interfaces.

[68] Pai C-F (2012). Spin transfer torque devices utilizing the giant spin Hall effect of tungsten. Appl. Phys. Lett..

[69] Mellnik AR (2014). Spin-transfer torque generated by a topological insulator. Nature.

[70] Lin X (2022). Strong enhancement of spin-orbit torques in ferrimagnetic Pt_*x*_(Si_3_N_4_)_1–*x*_/CoTb bilayers by Si_3_N_4_ doping. Phys. Rev. B.

[71] Gray I (2019). Spin seebeck imaging of spin-torque switching in antiferromagnetic Pt/NiO heterostructures. Phys. Rev. X.

[72] Baldrati L (2019). Mechanism of Néel order switching in antiferromagnetic thin films revealed by magnetotransport and direct imaging. Phys. Rev. Lett..

[73] DuttaGupta S (2020). Spin-orbit torque switching of an antiferromagnetic metallic heterostructure. Nat. Commun..

[74] Zhang P (2022). Control of Néel vector with spin-orbit torques in an antiferromagnetic insulator with tilted easy plane. Phys. Rev. Lett..

[75] Kang J (2020). Current-induced manipulation of exchange bias in IrMn/NiFe bilayer structures. Nat. Commun..

[76] Li J (2019). Spin Seebeck effect from antiferromagnetic magnons and critical spin fluctuations in epitaxial FeF_2_ films. Phys. Rev. Lett..

