# Supplementary material for: Strong variation of spin-orbit torques with relative spin relaxation rates in ferrimagnets
Source: Nat Commun. 2023 Mar 30;14:1778. doi: 10.1038/s41467-023-37506-9 (PMC10063689; doi:10.1038/s41467-023-37506-9)
Supplement: Supplementary file 1 — Supplementary Information [file 41467_2023_37506_MOESM1_ESM.pdf]

Supplementary Information for

# Strong variation of spin-orbit torques with relative spin relaxation rates in ferrimagnets

Lijun Zhu[1,2*] and Daniel C. Ralph[3,4]

1. *State Key Laboratory of Superlattices and Microstructures, Institute of Semiconductors, Chinese Academy of Sciences, Beijing 100083, China*

2. *College of Materials Science and Opto-Electronic Technology, University of Chinese Academy of Sciences, Beijing 100049, China*

3. *Cornell University, Ithaca, New York 14850, USA*

4. *Kavli Institute at Cornell, Ithaca, New York 14850, USA*

*[*ljzhu@semi.ac.cn](mailto:ljzhu@semi.ac.cn)*

**Contents:**

Supplementary Note 1. More details on magnetic properties of the $Fe_xTb_{1-x}$ samples

Supplementary Note 2. Harmonic Hall voltage response measurements

Supplementary Note 3. Field-like spin-orbit torque for the perpendicular magnetic anisotropy $Pt_{0.75}Ti_{0.25}/Fe_xTb_{1-x}$ bilayers

Supplementary Note 4. Spin- pumping enhancement of ferromagnetic resonance linewidth and damping

Supplementary Note 5. Estimation of the momentum scattering time of $Fe_xTb_{1-x}$

Supplementary Note 6. Negligible bulk spin-orbit torque in the 8 nm $Fe_xTb_{1-x}$

Supplementary Note 7. Temperature dependence of the spin Hall effect in $Pt_{0.75}Ti_{0.25}$

Supplementary Note 8. Mechanism of the temperature dependence of the resistivity of the 8 nm $Fe_{0.59}Tb_{0.41}$

## Supplementary Note 1. More details on magnetic properties of the $Fe_xTb_{1-x}$ samples

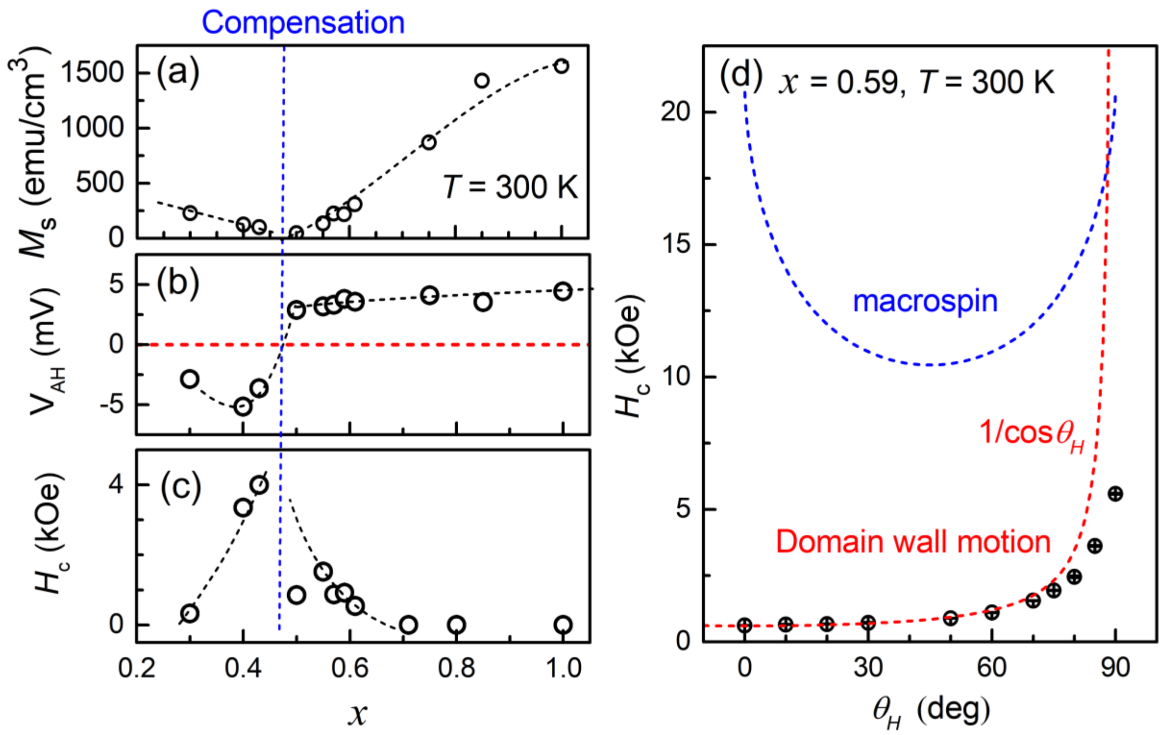


**Supplementary Fig. S1**. (a) Saturation magnetization, (b) Anomalous Hall voltage, (c) Coercivity of the $Pt_{0.75}Ti_{0.25}$ 5.6 nm/$Fe_xTb_{1-x}$ 8 nm bilayers, suggesting that the magnetization is fully compensated at the Fe concentration of $0.43 < x < 0.5$. (d) Coercivity ($H_c$) for the $Pt_{0.75}Ti_{0.25}$ 5.6 nm/$Fe_{0.59}Tb_{0.41}$ 8 nm plotted as a function of the polar angle of applied magnetic field ($\theta_H$), showing a significant deviation from the expectation for a macrospin [$H_c = H_k\,(\cos^{2/3}\theta_H + \sin^{2/3}\theta_H)^{-3/2}$, the dashed blue line], while it is relatively consistent with domain wall depinning ($\propto 1/\cos\theta_H$, solid red line). The $H_c$ values are determined from first harmonic Hall voltage hysteresis with the external field swept in the *xz* plane at different fixed polar angle of $\theta_H$.

As shown in Fig. S1a-c, for Fe concentrations near 0.5, the saturation magnetization of the $Pt_{0.75}Ti_{0.25}$ 5.6 nm/$Fe_xTb_{1-x}$ 8 nm bilayers diminishes, the anomalous Hall voltage reverses sign, and the coercivity appears to be overall enhanced. This together suggests that the magnetization is fully compensated at the Fe concentration slightly smaller than 0.5. The Fe concentration here is in volumetric percentage, not atomic percentage; it is also common that the magnetic properties of thin films of ferrimagnetic alloys can vary depending on growth protocols, substrate choices, and the layer thicknesses.

We then clarify that, while the samples for this study do exhibit a macrospin dynamics for small-angle tilting during the harmonic Hall voltage response measurements (see below), the magnetic field switching is governed mainly by domain wall depinning. If the thin magnetic layer followed Stoner-Wohlfarth macrospin behavior,[1] $H_c$, should be close to $H_k$ at the field polar angle of $\theta_H = 0^o$ in any finite measurement time, and $H_c$ should vary with $H_k\,(\cos^{2/3}\theta_H + \sin^{2/3}\theta_H)^{-3/2}$. However, the observed $H_c$ of the $Fe_xTb_{1-x}$ is much smaller than $H_k$ and significantly deviates from the scaling $H_k\,(\cos^{2/3}\theta_H + \sin^{2/3}\theta_H)^{-3/2}$ (Fig. S1d). Instead, $H_c$ much more closely follows a $1/\cos\theta_H$ scaling, as predicted by the switching via thermally-assisted reversed domain nucleation and domain wall propagation[2-4]. Therefore, the coercivity of micrometer-sized $Fe_xTb_{1-x}$ under applied perpendicular magnetic field is determined by the perpendicular depinning field.

## Supplementary Note 2. Harmonic Hall voltage response measurements

The spin-orbit-torque efficiencies of the $Pt_{0.75}Ti_{0.25}/Fe_xTb_{1-x}$ samples are determined using harmonic Hall voltage response (HHVR) measurements under a sinusoidal electric field $E$. For the perpendicular magnetic anisotropy samples ($x \leq 0.65$), the damping-like (field-like) spin-orbit torque effective field is given by [5]

$$H_{\mathrm{DL(FL)}} = -2\frac{\partial V_{2\omega}}{\partial H_{x(y)}} / \frac{\partial^2 V_{1\omega}}{\partial H^2_{x(y)}}, \qquad \text{(S1)}$$

where the in-phase first HHVR ($V_{1\omega}$) and the out-of-phase second HHVR ($V_{2\omega}$) are parabolic and linear functions of in-plane magnetic fields $H_{x(y)}$ (Fig. S2a,b), respectively. During the measurements, we first apply a large out-of-plane magnetic field to saturate the sample to avoid any intermediate or multi-domain states, and then record HHVR while sweeping $H_{x(y)}$ only in the small-field region (≤ 3.5 kOe). The planar Hall effect of the $Pt_{0.75}Ti_{0.25}/Fe_xTb_{1-x}$ samples in this work is negligibly small compared to the anomalous Hall effect anyway ($V_{\mathrm{PH}}/V_{\mathrm{AH}} < 0.086$, Fig. S3). As expected, $H_{\mathrm{DL}}$ is linearly proportional to $E$ (Fig. S2c).

We find that the out-of-plane HHVR measurements in this work are not affected by any thermoelectric effect. As indicated by the negligible second HHVR at very high fields (Fig. S4), there is negligible anomalous Nernst voltage associated with any vertical thermal gradient during our HHVR measurements. Note that the data in Fig. S4 were collected by sweeping the magnetic field along the in-plane current direction between ± 9 T. The slopes in Fig. S4 at low fields might therefore be affected by non-uniform, multi-domain magnetic states and should not be used to estimate the values of $H_{\mathrm{DL(FL)}}$.

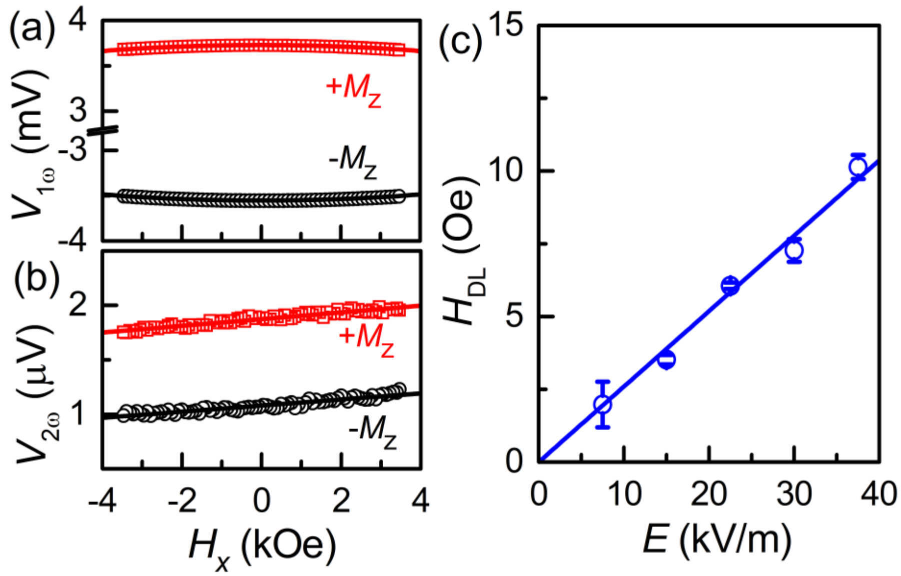


**Supplementary Fig.** S2. Out-of-plane HHVR measurement. (a) First HHVR ($V_{1\omega}$) *vs*. in-plane field $H_x$ and (b) Second HHVR ($V_{2\omega}$) *vs*. $H_x$ for a perpendicularly-magnetized $Pt_{0.75}Ti_{0.25}$ (5.6 nm)/$Fe_{0.59}Tb_{0.41}$ (8 nm) bilayer, for an applied electric field ($E$) of 30 kV/m. (c) Linear dependence of the damping-like SOT effective field ($H_{\mathrm{DL}}$) on $E$ for the $Pt_{0.75}Ti_{0.25}$ (5.6 nm)/$Fe_{0.59}Tb_{0.41}$ (8 nm) sample. The solid lines in (a) represent parabolic fits ($V_{1\omega} = \pm V_{\mathrm{AH}}(1-H_x^2/2H_k^2)$, from these fits the effective perpendicular anisotropic field $H_k$ is determined), while the solid lines in (b) and (c) are linear fits. The $E$ values in (c) are determined together by the input voltage, the length of the Hall bar device, and the contact resistances.

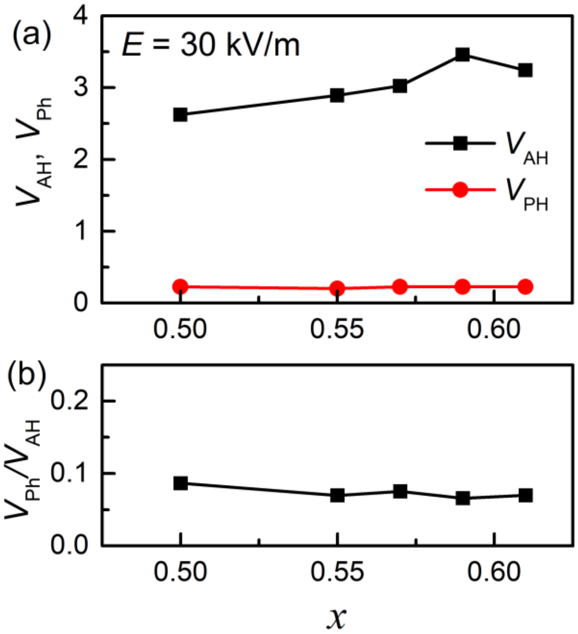


**Supplementary Fig.** S3. Comparison of the planar Hall voltage and the anomalous Hall effect for the Fe-rich perpendicularly-magnetized $Pt_{0.75}Ti_{0.25}$ (5.6 nm)/$Fe_{0.59}Tb_{0.41}$ (8 nm) bilayers.

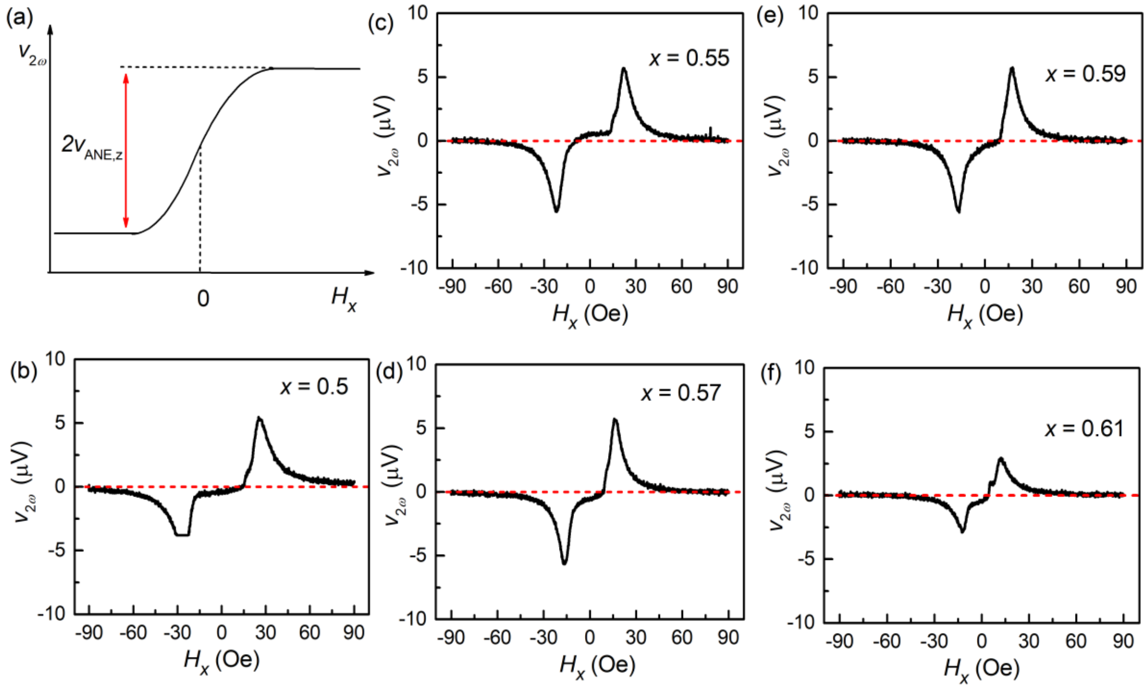


**Supplementary Fig.** S4. Demonstration of negligible anomalous Nernst voltage for the measurements on PMA samples. (a) Schematic of the expected second HHVR ($V_{2\omega}$) contribution from an anomalous Nernst voltage. When the perpendicular magnetization is aligned parallel and antiparallel to the in-plane current direction by a magnetic field that is greater than anisotropic field, the difference of the $V_{2\omega}$ values is two times the anomalous Nernst voltage. (b-f) Dependence of $V_{2\omega}$ on applied in-plane magnetic field $H_x$ for the representative $Pt_{0.75}Ti_{0.25}$ (5.6 nm)/$Fe_xTb_{1-x}$ (8 nm) samples with the Fe concentrations of (b) 0.5, (c) 0.55, (d) 0.57, (e) 0.59, and (f) 0.61. The red dashed lines indicate the zero-voltage position. The electric field ($E$) is 30 kV/m.

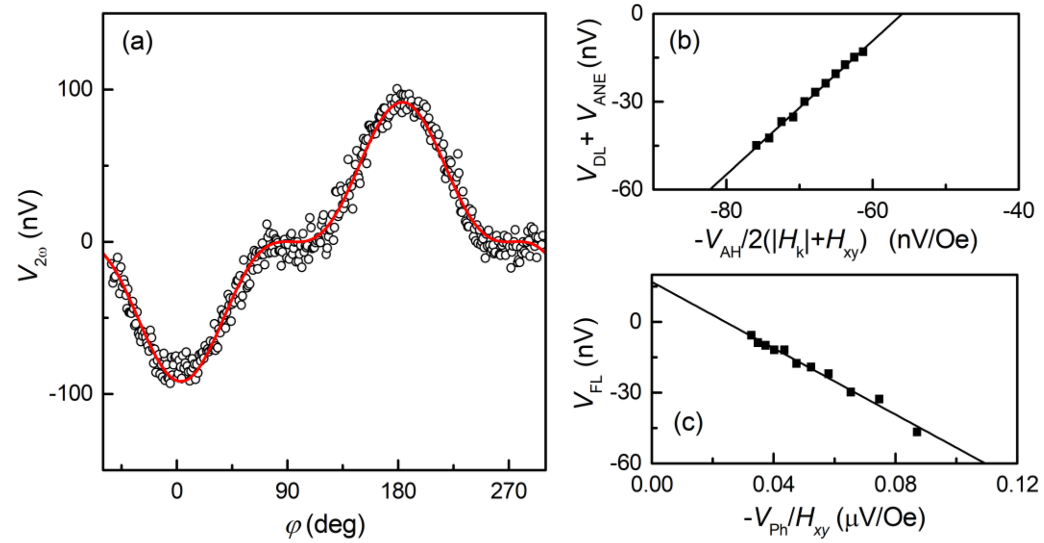


**Supplementary Fig.** S5. In-plane HHVR measurement. (a) Second HHVR ($V_{2\omega}$) *vs*. in-plane angle of magnetic field $H_{xy}$ with respect to the current direction, (b) $V_{DL}$ *vs*. $-V_{AH}/2(H_{xy}+|H_k|)$, and (c) $V_{FL}$ *vs*. $V_{PH}/H_{xy}$ for a $Pt_{0.75}Ti_{0.25}$ (5.6 nm)/$Fe_{0.85}Tb_{0.15}$ (8 nm) sample. The excitation electric field is $E$ = 30 kV/m.

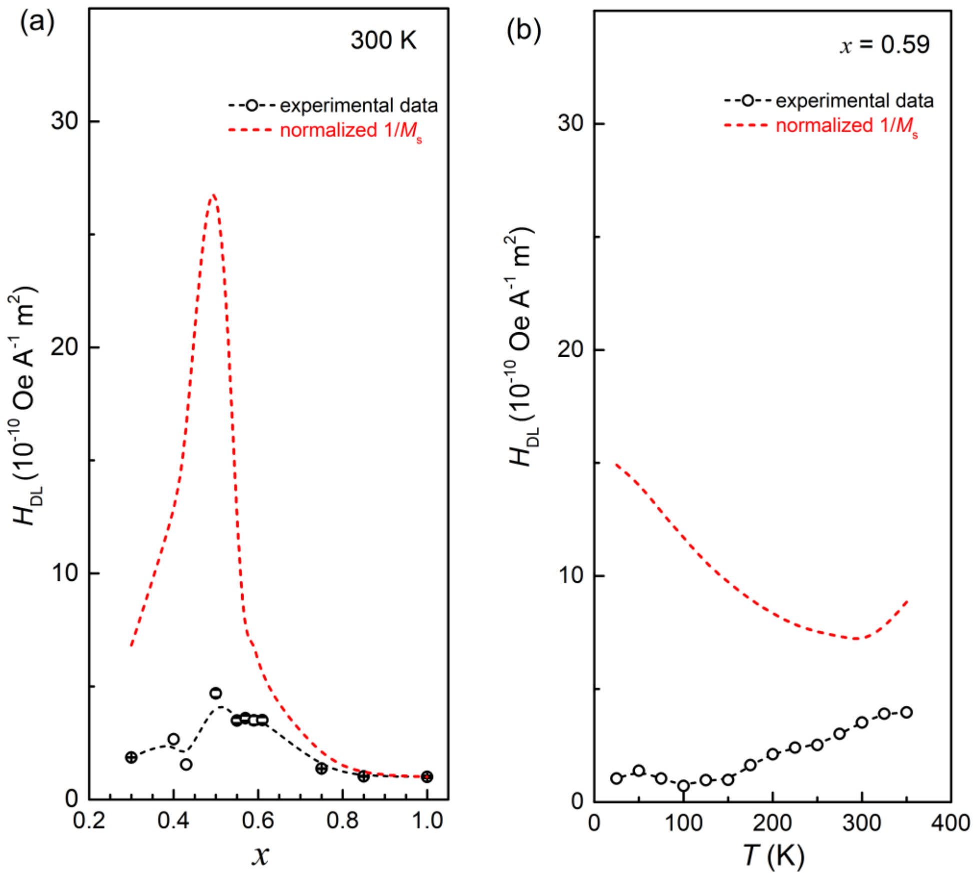


**Supplementary Fig. S6**. Damping-like effective spin-orbit torque field ($H_{DL}$) of $Pt_{0.75}Ti_{0.25}$ (5.6 nm)/$Fe_xTb_{1-x}$ (8 nm) bilayers measured for (a) for the composition series ($T$ = 300 K, $x$ = 0.3-1) and for (b) the temperature series ($T$ = 25-350 K, $x$ = 0.59). The experimental data of $H_{DL}$ clearly deviates from the normalized inverse magnetization $1/M_s$ (the red dashed lines) which diverges upon approaching the magnetization compensation points.

For the in-plane-magnetized samples ($x \geq 0.75$), $H_{DL}$ is determined from angle-dependent in-plane HHVR measurements. In this case, the second HHVRs are collected by sweeping the angle ($\varphi$) of the in-plane magnetic field $H_{xy}$ and thus the magnetization with respect to the current direction. The magnitudes of $H_{xy}$ are 1 kOe to 8 kOe. As shown in Fig. S5a, $V_{2\omega}$ follows $V_{2\omega} = (V_{DL}+ V_{ANE}) \cos\varphi + V_{FL} \cos\varphi\cos2\varphi$, where $V_{DL} = - V_{AH}H_{DL}/2(H_{xy}+|H_k|)$ is the second HHVR for the damping-like SOT, $V_{FL} = V_{PH} (H_{FL} + H_{Oe})/H_{xy}$ the second HHVR for the field-like SOT and Oersted field torque, $V_{ANE}$ the anomalous Nernst voltage, $H_{xy}$ the in-plane bias field, and $H_k$ the effective perpendicular anisotropy field as measured from the saturation field of the hard axis. $H_{DL}$ is determined from a linear fit of $V_{DL}$ *vs* - $V_{AH}/2(H_{xy}+|H_k|)$ and $H_{FL}$ is determined from a linear fit of $V_{FL}$ *vs*. $V_{PH}/H_{xy}$ (Fig. S5b,c).

As shown in Fig. S6a,b, $H_{DL}$ of the $Pt_{0.75}Ti_{0.25}$ (5.6 nm)/$Fe_xTb_{1-x}$ (8 nm) does *not* scale linearly with $1/M_s$ or diverge upon approaching the magnetization compensation points in the composition series and the temperature series. This reveals that the damping-like SOT efficiency ($\xi_{DL}^j$) is not constant but rather varies with composition and temperature.

## Supplementary Note 3. Field-like spin-orbit torque for the perpendicular magnetic anisotropy $Pt_{0.75}Ti_{0.25}/Fe_xTb_{1-x}$ bilayers

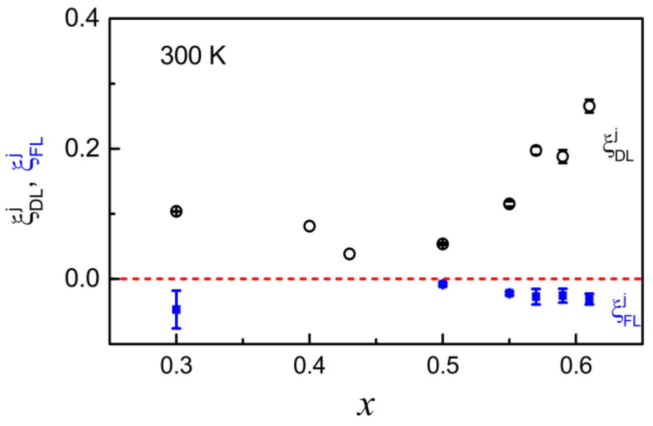


**Supplementary Fig. S7**. Efficiencies of the damping-like ($\xi_{DL}^j$) and field-like SOT ($\xi_{FL}^j$) of the perpendicular magnetic anisotropy $Pt_{0.75}Ti_{0.25}$ (5.6 nm)/$Fe_xTb_{1-x}$ (8 nm) bilayers with different Fe concentrations $x$. $\xi_{FL}^j$ is smaller than $\xi_{DL}^j$ and varies with $x$.

## Supplementary Note 4. Spin- pumping enhancement of ferromagnetic resonance linewidth and damping

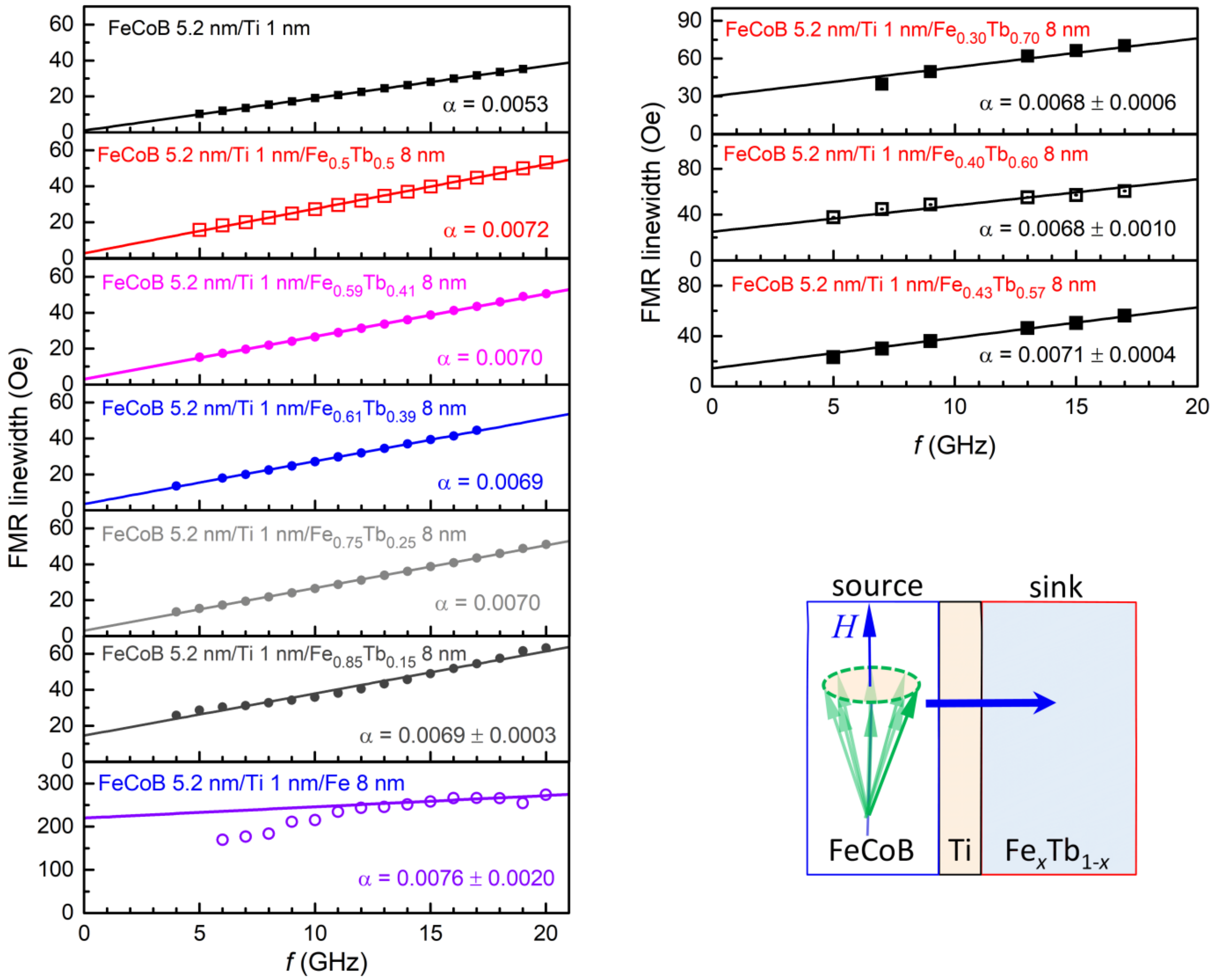


**Supplementary Fig. S8**. Frequency dependence of the ferromagnetic resonance linewidth ($\Delta H$) of the FeCoB layer in FeCoB (5.2 nm)/Ti (1 nm) and FeCoB (5.2 nm)/Ti (1 nm)/$Fe_xTb_{1-x}$ (8 nm) samples with $x$ =0.5, 0.59, 0.61, 0.75, 0.85, 1, 0.3, 0.4, and 0.43. The solid lines represent linear fits, the slopes of which yield the damping parameter ($\alpha$). The damping of FeCoB (5.2 nm)/Ti (1 nm)/$Fe_xTb_{1-x}$ (8 nm) samples is around 0.007, regardless of the $Fe_xTb_{1-x}$ composition. This observation indicates that that the effective spin mixing conductance ($G_{\mathrm{eff}}^{\uparrow\downarrow}$) of the FeCoB/Ti (1 nm)/$Fe_xTb_{1-x}$ interface is not affected by magnetic compensation for $Fe_xTb_{1-x}$.

## Supplementary Note 5. Estimation of the momentum scattering time of $Fe_xTb_{1-x}$

We make a rough estimate of the momentum-scattering time ($\tau_e$) of the $Fe_xTb_{1-x}$ from the resistivity of the $Fe_xTb_{1-x}$ following the Drude model $\rho_{\mathrm{FeTb}} = m^*/ne^2\tau_e$, where $m^*$ and $n$ are the effective mass and the density of the charge carriers. Previous experiments[6] and theories[7] have suggested that the conduction of the disordered $Fe_xTb_{1-x}$ is dominated by holes of the $d$ bands and that the Drude model provides a realistic description for the relation of resistivity and $n$ of the disordered $Fe_xTb_{1-x}$. Since both Fe and Tb contribute $d$ states to the Fermi surface of $Fe_xTb_{1-x}$, $m^*$ of the $d$ holes might vary slightly with temperature and composition but that should not alter the qualitative variation of $\tau_e$ we estimate below.

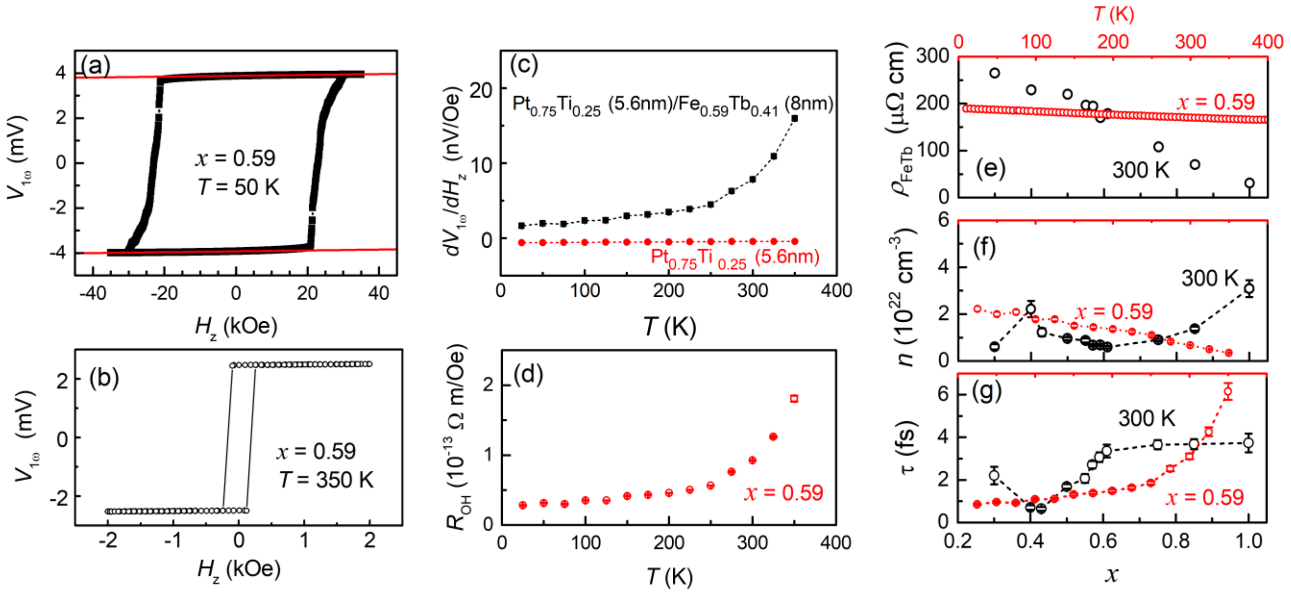


**Supplementary Fig. S9**. (a,b) First HHVR ($V_{1\omega}$) *vs* out-of-plane magnetic field ($H_z$) for a $Pt_{0.75}Ti_{0.25}$ (5.6 nm)/$Fe_{0.59}Tb_{0.41}$ (8 nm) bilayer at 50 K and 350 K, respectively. The two red lines in (a) represent linear fits. (c) Slopes of the linear fits of $V_{1\omega}$ *vs* $H_z$, i.e., $dV_{1\omega}/dH_z$ in high magnetic field regimes for $Pt_{0.75}Ti_{0.25}$ (5.6 nm)/$Fe_{0.59}Tb_{0.41}$ (8 nm) and $Pt_{0.75}Ti_{0.25}$ (5.6 nm). In (a)-(c) the electric field $E$ is 30 kV/m. (d) Ordinary Hall coefficient *vs* temperature for the $Fe_{0.59}Tb_{0.41}$ (8 nm) bilayer. (e) Resistivity, (f) carrier density, and (g) momentum scattering time for $Fe_xTb_{1-x}$ in the composition series (black circles, $T$ = 300 K, $x$ = 0.3-1) and the temperature series (red circles, $T$ = 25-350 K, $x$ = 0.59).

We first measured the first-harmonic Hall response voltage ($V_{1\omega}$) of the $Fe_xTb_{1-x}$ as a function of out-of-plane field ($H_z$). We then measured the slope of the linear fit of $V_{1\omega}$ *vs* $H_z$, i.e., $dV_{1\omega}/dH_z$ (Fig. S9a,b) in the high field regime for the $Pt_{0.75}Ti_{0.25}$ 5.6 nm/$Fe_xTb_{1-x}$ 8 nm bilayers and for a 5.6 nm $Pt_{0.75}Ti_{0.25}$ control sample by applying the same electric field $E$. As shown in Fig. S9, $dV_{1\omega}/dH_z$ of the 5.6 nm $Pt_{0.75}Ti_{0.25}$ shows little temperature dependence and is negligibly small compared to that of the $Pt_{0.75}Ti_{0.25}$ 5.6 nm/$Fe_xTb_{1-x}$ 8 nm bilayers, suggesting that the $dV_{1\omega}/dH_z$ values of the $Pt_{0.75}Ti_{0.25}$ 5.6 nm/$Fe_xTb_{1-x}$ 8 nm bilayers are dominantly contributed by the ferrimagnetic $Fe_xTb_{1-x}$ layer at all temperatures. After subtracted the small $dV_{1\omega}/dH_z$ contribution from that of the $Pt_{0.75}Ti_{0.25}$ 5.6 nm/$Fe_xTb_{1-x}$ 8 nm bilayers, we obtain the $dV_{1\omega}/dH_z$ values for the $Fe_xTb_{1-x}$ layers. The ordinary Hall coefficient ($R_{OH}$) of the $Fe_xTb_{1-x}$ is then determined following the relation $R_{OH} = (\rho_{xx}/EW)\, dV_{1\omega}/dH_z$, where $\rho_{xx}$ is the resistivity of the $Fe_xTb_{1-x}$ and $W$ is the width of the Hall bar.

As shown in Fig. S10d, $R_{OH}$ of the $Fe_xTb_{1-x}$ increases strongly with raising temperature, which is consistent with previous reports of strong variation of $R_{OH}$ with temperature in Fe and Tb due to temperature tuning of Fermi surface properties[8,9]. Note that at high magnetic fields, the Hall voltages of the $Pt_{0.75}Ti_{0.25}$/$Fe_xTb_{1-x}$ samples are fairly good linear function of magnetic field in the whole studied temperature range, suggesting a magnetic-field-independent ordinary Hall coefficients (see Fig. S10b for the 350 K data for the $Pt_{0.75}Ti_{0.25}$ /$Fe_{0.59}Tb_{0.41}$). The increase of $R_o$ in the whole temperature region cannot be explained by any effects due to approaching the Curie temperature (400 K for the $Pt_{0.75}Ti_{0.25}$ /$Fe_{0.59}Tb_{0.41}$).

Using the values of $R_{OH}$ (Fig. S9d) and the relation $R_{OH} = 1/ne$ for a single-band model, we estimated the charge carrier densities of the $Fe_xTb_{1-x}$ layers (Fig. S9g). As discussed in Ref. 8, the single-band model $R_{OH} = 1/ne$ should be accurate for less-pure samples, such as our sputter-deposited disordered $Fe_xTb_{1-x}$ that is either polycrystalline or amorphous. We find that $n$ varies by a factor of 4 for $Fe_xTb_{1-x}$ as a function of composition, as by a factor of 7 for $Fe_{0.59}Tb_{0.41}$ as a function of temperature. As suggested by the significant variations in the anomalous Hall voltage of $Fe_xTb_{1-x}$ as a function of composition and temperature (Fig. S10), the Fermi surface properties of the $Fe_xTb_{1-x}$ are strongly tuned by the composition and temperature.

The competition of the Tb 5*d* and 6*s* electrons and the Fe 3*d* and 4*s* electrons at the Fermi surface appears to explain the moderate and non-monotonic change of the carrier density as a function of the composition of $Fe_xTb_{1-x}$. Rigid understanding of the variations of the carrier density requires precise calculation of the band structure for the amorphous $Fe_xTb_{1-x}$ and is beyond the scope of our present work. With the values of $n$ and $\rho_{FeTb}$ in Figs. S9c,d, the values of $\tau_e$ are estimated and plotted in Fig. S9e and Fig. 3b of the main text.

Finally, we note that the applicability of a single-band model for the ordinary Hall effect for estimating $\tau_e^{-1}$ is not essential for our conclusions of the strong variation of $\xi_{DL}^{j}$ with relative spin relaxation rates, since similar scaling in Fig. 3e of the main text is present even when simply plotting $\xi_{DL}^{j}$ as a function of $M_s/\zeta_{so}\rho_{xx}$, see Fig. S11.

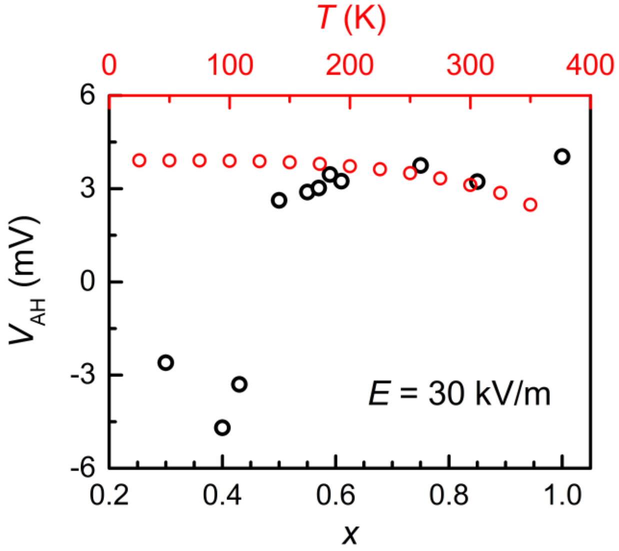


**Supplementary Fig. S10**. Anomalous Hall voltage for $Fe_xTb_{1-x}$ for the composition series (black circles, $T$ = 300 K, $x$ = 0.3-1) and for the temperature series (red circles, $T$ = 25-350 K, $x$ = 0.59). While the actual strengths of the electric bias field varied slightly for different samples due to variation of contact resistances during the measurements, we have rescaled the anomalous Hall voltages so that the values in the plot correspond to the same bias field of 30 kV/m.

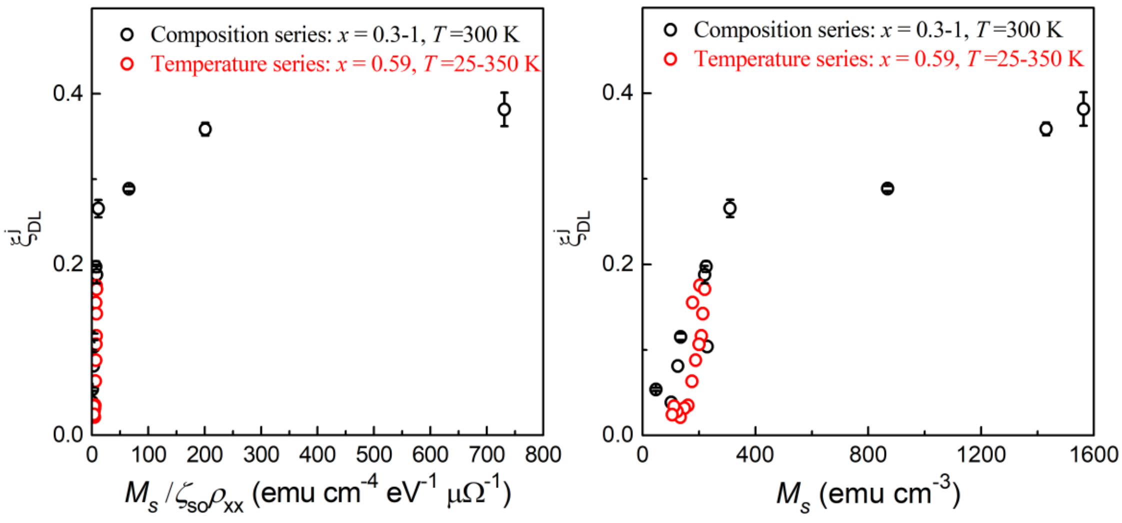


**Supplementary Fig. S11**. Damping-like SOT efficiency ($\xi_{DL}^{j}$) of $Pt_{0.75}Ti_{0.25}/Fe_xTb_{1-x}$ vs $M_s/\zeta_{so}\rho_{xx}$ and $\xi_{DL}^{j}$ vs $M_s$ for the composition series ($x$ = 0.3-1, $T$ = 300 K, black circles) and for the temperature series ($x$ = 0.59, $T$ = 25-300 K, red circles).

**Supplementary Note 6. Negligible bulk spin-orbit torque in the 8 nm $Fe_xTb_{1-x}$**

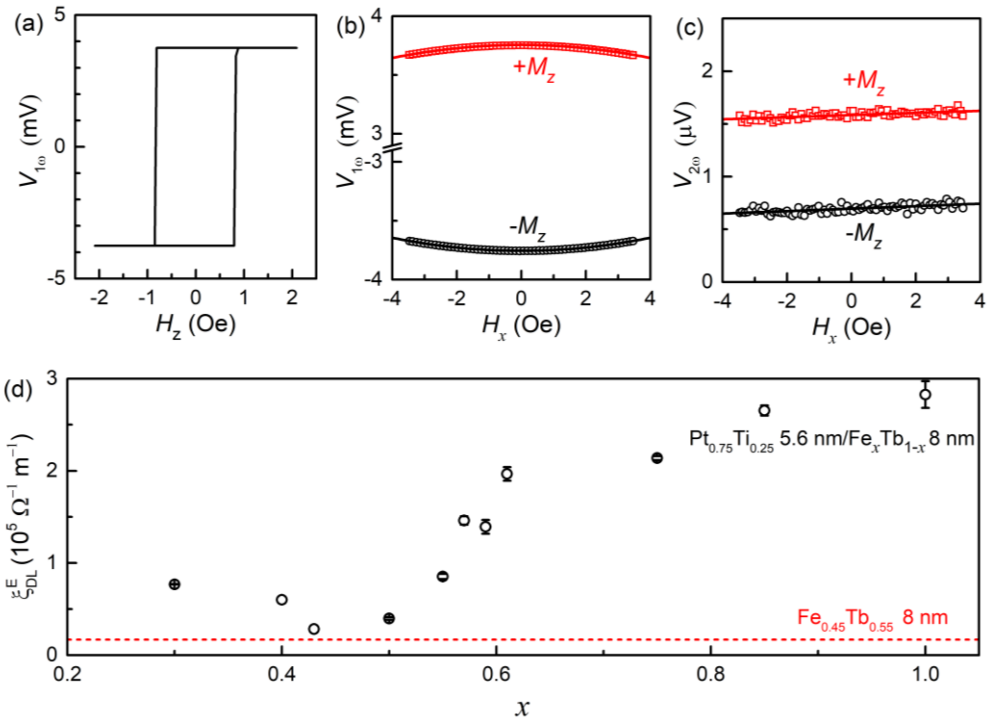


**Fig. S12** Out-of-plane HHVR measurement on 8 nm $Fe_{0.55}Tb_{0.45}$ single layer. (a) First HHVR ($V_{1\omega}$) *vs*. out-of-plane field $H_z$, (b) $V_{1\omega}$ *vs*. in-plane field $H_x$ and (c) Second HHVR ($V_{2\omega}$) *vs*. $H_x$ for an applied electric field ($E$) of 30 kV/m. The solid lines in (b) represent parabolic fits ($V_{1\omega} = \pm V_{AH}(1\text{-}H_x^2/2H_k^2)$, while the solid lines in (c) are linear fits. (d) Comparison of damping-like SOT efficiency per electric field for the 8 nm $Fe_{0.55}Tb_{0.45}$ single layer and for the $Pt_{0.75}Ti_{0.25}$ 5.6 nm/$Fe_xTb_{1-x}$ 8 nm bilayers.

To evaluate possible bulk SOT in the 8 nm $Fe_xTb_{1-x}$ layers, we perform HHVR measurements on a control sample of 8 nm $Fe_{0.55}Tb_{0.45}$ single layer with strong perpendicular magnetic anisotropy and square anomalous Hall voltage hysteresis (Fig. S12a, the complete stack of Si/$SiO_2$/Ta 1 nm/ $Fe_{0.55}Tb_{0.45}$ 8 nm/MgO 2/Ta 1.5 nm). The composition $Fe_{0.55}Tb_{0.45}$ is chosen because we recently find that $Fe_xTb_{1-x}$ layers with large thicknesses of 20-90 nm exhibit a bulk SOT that has peaked strength at the composition around $Fe_{0.55}Tb_{0.45}$. From high-quality HHVR data from the 8 nm $Fe_{0.55}Tb_{0.45}$ (Fig. S12b,c), we determine a spin-orbit torque field $H_{DL}$ = 1.57±0.11 Oe under $E$ = 30 kV/m. Here, we do not calculate the SOT efficiency per current density $\xi^j_{DL} = (2e/\hbar)M_s t_{FeTb} H_{DL}/j_c$ for the $Fe_{0.55}Tb_{0.45}$ single layer because $j_c$ is the charge current density of the $Pt_{0.75}Ti_{0.25}$ layer in the torque analysis of the $Pt_{0.75}Ti_{0.25}$ 5.6 nm/ $Fe_xTb_{1-x}$ 8 nm bilayers in the main text. Instead, we calculate damping-like SOT efficiency per electric field of $\xi^E_{DL} = (2e/\hbar)M_s t_{FeTb} H_{DL}/E$, which provides a comparison of the torque contributions of the $Pt_{0.75}Ti_{0.25}$ and the $Fe_xTb_{1-x}$ on the same footing. Since $\xi^j_{DL} = \xi^E_{DL}\rho_{xx}$ for the $Pt_{0.75}Ti_{0.25}$/$Fe_xTb_{1-x}$ bilayers in the main text, a nonzero bulk SOT could contribute to $\xi^E_{DL}$ and then affect the calculated values of $\xi^j_{DL}$.

From the HHVR measurement, we determine that the 8 nm $Fe_{0.55}Tb_{0.45}$ only contributes a negligibly small $\xi^E_{DL}$ of (0.17 ± 0.01)×$10^5$ $\Omega^{-1}$ $m^{-1}$. As we compare in Fig. S12d, this $\xi^E_{DL}$ is a factor of 5 smaller than the torque for the $Pt_{0.75}Ti_{0.25}$ 5.6 nm/$Fe_{0.55}Tb_{0.45}$ 8 nm and even less than the "vanishingly small" torque of the $Pt_{0.75}Ti_{0.25}$ 5.6 nm/$Fe_xTb_{1-x}$ 8 nm bilayers with $x$ =0.5 and 0.43. Since the negligibly small $\xi^E_{DL}$ for the 8 nm $Fe_{0.55}Tb_{0.45}$ single layer is supposed to already represent the highest possible damping-like SOT efficiency that the 8 nm $Fe_xTb_{1-x}$ can contribute, we can conclude that there is only negligible bulk SOT from the 8 nm $Fe_xTb_{1-x}$ in this work. This is consistent with previous observation that a bulk torque of a magnetic layer is strongly thickness dependent and vanishes at small layer thickness of a few nanometers[9].

## Supplementary Note 7. Temperature dependence of the spin Hall effect in $Pt_{0.75}Ti_{0.25}$

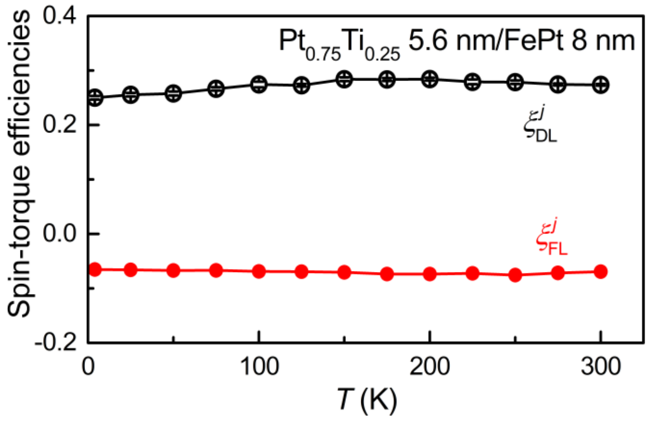


**Supplementary Fig. S13** Temperature dependence of damping-like and field-like spin-orbit torque efficiencies per current density, $\xi_{DL}^{j}$ and $\xi_{FL}^{j}$, for ferrimagnetic $Pt_{0.75}Ti_{0.25}$ 5.6 nm/FePt 8 nm, suggesting that the spin-orbit torque efficiencies and thus the spin Hall effect of the $Pt_{0.75}Ti_{0.25}$ only have a very weak dependence on the temperature.

## Supplementary Note 8. Mechanism of the temperature dependence of the resistivity of the 8 nm $Fe_{0.59}Tb_{0.41}$

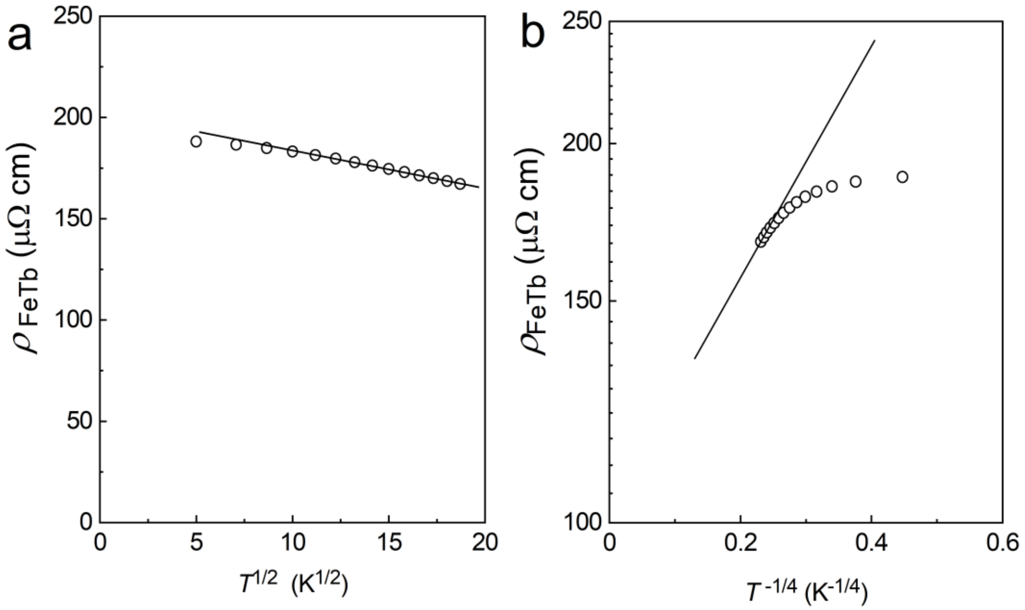


**Supplementary Fig. S14 Temperature ($T$) dependence of the resistivity ($\rho_{FeTb}$) of the 8 nm $Fe_{0.59}Tb_{0.41}$.** (a) $\rho_{FeTb}$ vs $T^{1/2}$, suggesting that the resistivity varies linearly with $T^{1/2}$, in good agreement with the mechanism of electron-electron interaction.[10] (b) Log plot of $\rho_{FeTb}$ as a function of $T^{-1/4}$, indicating a considerable deviation from the Mott's law for hopping conduction,[11] the latter predicts $\ln\rho_{FeTb}$ to vary linearly with $T^{-1/4}$.

### References


1. E. C. Stoner, E. P. Wohlfarth, A mechanism of magnetic hysteresis in heterogeneous alloys, Philos. Trans. R. Soc. **240**, 599 (1948).
2. F. Schumacher, On the modification of the Kondorsky function, J. Appl. Phys. **70**, 3184 (1991).

3. O. J. Lee, L. Q. Liu, C. F. Pai, Y. Li, H. W. Tseng, P. G. Gowtham, J. P. Park, D. C. Ralph, and R. A. Buhrman, Central role of domain wall depinning for perpendicular magnetization switching driven by spin torque from the spin Hall effect, Phys. Rev. B 89, 024418 (2014).
4. L. Zhu, D.C. Ralph, R.A. Buhrman, Lack of Simple Correlation between Switching Current Density and Spin-Orbit-Torque Efficiency of Perpendicularly Magnetized Spin-Current-Generator–Ferromagnet Heterostructures, Phys. Rev. Appl. 15, 024059 (2021).
5. L. J. Zhu, K. Sobotkiewich, X. Ma, X. Li, D. C. Ralph, R. A. Buhrman, Strong Damping-Like Spin-Orbit Torque and Tunable Dzyaloshinskii–Moriya Interaction Generated by Low-Resistivity $Pd_{1-x}Pt_x$ Alloys, Adv. Funct. Mater. 29, 1805822 (2019).
6. G. A. N. Connell and Dan S. Bloomberg, "Amorphous Rare-Earth Transition-Metal Alloys", Physics of Disordered Materials, edited by David Adler, Hellmut Fritzsche, Stanford R. Ovshinsky, Plenum Press 1985, pp 739-752.
7. G. T. Meaden, Conduction Electron Scattering and the Resistance of the Magnetic Elements, Contemp. Phys. 12, 313-337 (1971).
8. M.G. Cottam and R.B. Stinchcombe, The theory of the ordinary Hall coefficient of iron at low temperatures, J. Phys. C, 1, 1052 (1968).
9. J. J. Rhyne, Anomalous and Ordinary Hall Effect in Terbium, J. Appl. Phys. 40,1001 (1969).
10. L. Zhu, X.S. Zhang, D.A Muller, D.C. Ralph, R.A Buhrman, Observation of strong bulk damping-like spin-orbit torque in chemically disordered ferromagnetic single layers, Adv. Funct. Mater. 30, 2005201 (2020).
11. L. Zhu, J. H. Zhao, Anomalous resistivity upturn in epitaxial $L2_1$-$Co_2MnAl$ films, Sci. Rep. 7, 42931 (2017).
12. N. F. Mott, The metal-insulator transition in extrinsic semiconductors, Adv. Phys. 21, 785 (1972).
